# Copper nanoparticle exsolution from Sr(Ti, Fe)O_3_ perovskites: material tuning and probing (electro)catalytic applicability

**DOI:** 10.1039/d5na00426h

**Published:** 2025-12-10

**Authors:** Ubong Akpan Essien, Swathi Patchaiammal Raju, Keyla Teixeira Santos, Rafael Alcides Vicente, Chinyere Adaora Ekperechukwu, Francisco R. García-García, Pablo Sebastián Fernández, Dragos Neagu

**Affiliations:** a Strathclyde Incubator for Green Hydrogen Technology (SigH2t), Chemical and Process Engineering, University of Strathclyde 16 Richmond Street Glasgow G1 1XQ UK dragos.neagu@strath.ac.uk; b Department of Physical Chemistry, State University of Campinas (UNICAMP) Campinas 13083-970 Brazil; c School of Engineering, Institute for Materials and Processes, The University of Edinburgh Sanderson Building, Robert Stevenson Road Edinburgh EH9 3FB UK; d University of Edinburgh, Max Born Crescent, Alrick Building, Engineering Stores Edinburgh EH9 3BF UK

## Abstract

Copper (Cu) is a recyclable, abundant, and promising catalyst for energy transition reactions like electrochemical conversion of nitrate (NO_3_RR) and CO_2_ electroreduction. However, conventional Cu-based electrocatalysts struggle with activity, selectivity, and durability, especially under harsh electrochemical conditions. Exsolution—the *in situ* generation of metallic nanoparticles on oxide supports in a single step—enables tightly anchored, size-controlled particles, enhancing stability and performance. Incorporating Cu into Sr_1−*α*_(Ti, Fe)O_3−*γ*_ perovskites, an earth-abundant system with promising ionic–electronic conductivity and adequate oxygen vacancies, overcomes the limitations of traditional Sr(Ti, Fe)O_3−*γ*_ in facilitating nanoparticle exsolution. This work demonstrates controlled Cu nanoparticle exsolution from Sr_0.95_Ti_0.3_Fe_0.7−*x*_Cu_*x*_O_3−*γ*_ perovskites at temperatures as low as 400 °C, notably milder than conventional exsolution conditions. By systematically varying reduction parameters, we achieve control over nanoparticle size (13–38 nm) and population density (118–650 particles per µm^2^). Electrochemical characterisation using nitrate reduction as a probe reaction demonstrates how exsolution conditions directly influence surface reactivity, establishing these materials as tuneable platforms for (electro)catalytic applications.

## Abstract 2

Sr_0.95_Ti_0.3_Fe_0.6_Cu_0.1_O_3_ perovskite shows enhanced reducibility and controlled Cu nanoparticles exsolution under reduction. Cu-doping tunes exsolution kinetics, particle density, and nitrate-reduction electrocatalytic response.

## Graphical abstract

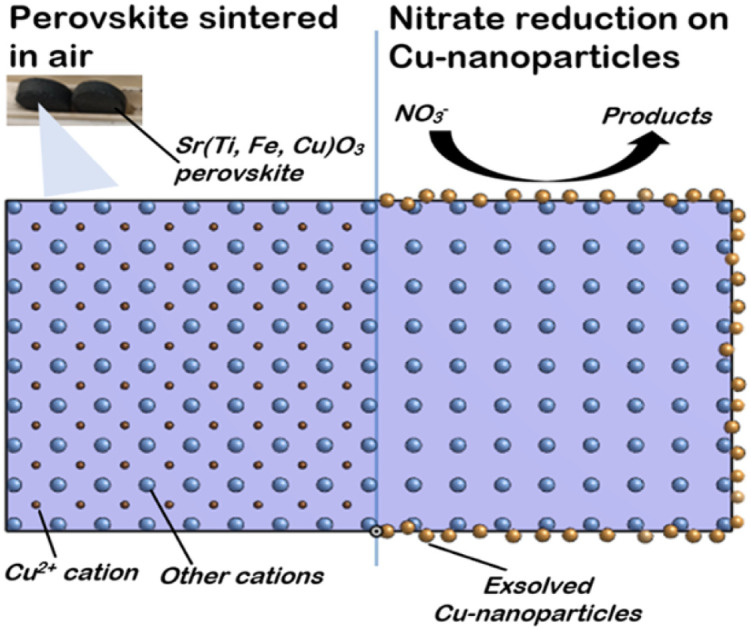

## Introduction

Developing sustainable technologies to ensure efficient energy conversion and storage is crucial to address the challenges associated with the long-term reliance on fossil fuels. In this context, copper-based catalysts have garnered significant attention due to their abundance, recyclability, and versatility in various applications. These include electrochemical nitrate reduction (NO_3_RR) for water remediation and ammonia production, hydrogen evolution reaction for green hydrogen production, and CO_2_ reduction for value-added molecules and platform chemicals, pivotal for energy transition and environmental sustainability.^1–6^ However, conventional Cu-based catalysts often lack activity, selectivity, and durability, particularly under the harsh conditions of electrochemical and thermochemical reactions. Innovative strategies that enhance catalyst stability, tunability, and performance are essential to overcome these limitations.

One approach involves incorporating copper (ions) into perovskite oxides, facilitating the exsolution of well-dispersed Cu (metal) nanoparticles at the surface.^7^ This technique allows for precise control over catalytic activity.

It addresses the challenges related to particle agglomeration and detachment during operation, paving the way for more robust and efficient catalytic systems. Moreover, the rational design of these catalysts is achievable by controlling key exsolution parameters such as temperature, time, and atmosphere, enabling the fine-tuning of nanoparticle size, shape, and surface distribution to meet specific application requirements.^8^

Perovskite oxides are materials with the ABO_3_ structure, where A and B represent cations while O represents an anion. This material has been used for several applications, mainly due to its tuneable structure, robust stability, and flexible doping capabilities, which allow fine-tuning of electronic and structural properties to optimise catalytic performance.^6^ Achieving Cu exsolution—a process where Cu ions segregate from the perovskite lattice to the surface to form well-anchored metallic nanoparticles—addresses common issues in catalysis by creating uniformly distributed stable nanoparticles that resist agglomeration and detachment. Adjusting reduction conditions, such as temperature and duration, allows the nanoparticles' size, shape, and density to be systematically controlled, offering significant advantages over traditional catalyst preparation methods.^9–12^

The success of enhanced exsolution is tied to selecting perovskites with desirable properties such as ionic and electronic conductivity, thermochemical stability, and the ability to form oxygen vacancies, all of which are critical for (electro)catalytic applications.^13–15^ Among non-rare-metal-based perovskites, STF oxides have shown particular promise. They offer mixed ionic and electronic conductivity along with suitable electrochemical stability.^16–18^ In particular, the Sr_1−*x*_Ti_1−*γ*_Fe_*γ*_O_3_ system has demonstrated chemical and structural robustness and adaptability for stoichiometric variation, allowing targeted doping with catalytically active elements like Cu.^19–22^

This study explores the rational design of Sr_0.95_Ti_0.3_Fe_0.7−*x*_Cu_*x*_O_3−*γ*_ by leveraging A-site deficiency and Cu-doping to achieve controlled exsolution of Cu nanoparticles at relatively low temperatures. This approach enabled the formation of well-dispersed Cu nanoparticles at reduction temperatures as low as 400 °C within 1 hour. By systematically varying the exsolution temperature and time, we demonstrate how these parameters influence nanoparticle properties and catalytic activity, providing a tunable platform for tailored applications. As a proof-of-concept and surface characterisation tool indicative of electrochemical application suitability, we applied the materials to the NO_3_RR, illustrating how the rational design of exsolution conditions can tune the electrochemical properties of the catalyst.

## Results and discussion

### Perovskite system design and synthesis conditions

The Sr_0.95_Ti_0.3_Fe_0.7−*x*_Cu_*x*_O_3−*γ*_ series of perovskites with varying Cu-doping (*x* = 0, 0.05, 0.1, 0.15, 0.2 mol) were designed to be A-site deficient, resulting in an A-to-B site ratio below 1, which promotes exsolution capability, as reported previously.^23,24^ The Cu doping range (*x* = 0–0.2) was selected to explore the limits of Cu substitution in this perovskite system. While higher Cu content would provide more potentially active sites, it would also introduce excessive oxygen vacancies due to charge compensation with host cations (Ti^4+^ and Fe^3+/4+^) and, given Cu's position as the most reducible first-row transition metal ion, would make the overall lattice more reducible.^25^ This could lead to uncontrolled structural decomposition rather than the desired controlled exsolution of nanoparticles. Table 1 presents the stoichiometry of the respective perovskites.

**Table 1:** Composition of the Sr_0.95_Ti_0.3_Fe_0.7−*x*_Cu_*x*_O_3−*γ*_ perovskite based on nominal Cu-doping amount in moles

Stoichiometry composition	Nominal Cu-doping amount	Nominal substitution/comment
Sr_0.95_Ti_0.3_Fe_0.7_O_3−*γ*_	0	5% A-site deficiency, B-site: 30% Ti, 70% Fe
Sr_0.95_Ti_0.3_Fe_0.65_Cu_0.05_O_3−*γ*_	0.05	5% A-site deficiency, B-site: 30% Ti, 65% Fe, 5% Cu
Sr_0.95_Ti_0.3_Fe_0.6_Cu_0.1_O_3−*γ*_	0.1	5% A-site deficiency, B-site: 30% Ti, 60% Fe, 10% Cu
Sr_0.95_Ti_0.3_Fe_0.55_Cu_0.15_O_3−*γ*_	0.15	5% A-site deficiency, B-site: 30% Ti, 55% Fe, 15% Cu
Sr_0.95_Ti_0.3_Fe_0.6_Cu_0.2_O_3−*γ*_	0.2	5% A-site deficiency, B-site: 30% Ti, 50% Fe, 20% Cu

**Fig. 1:**
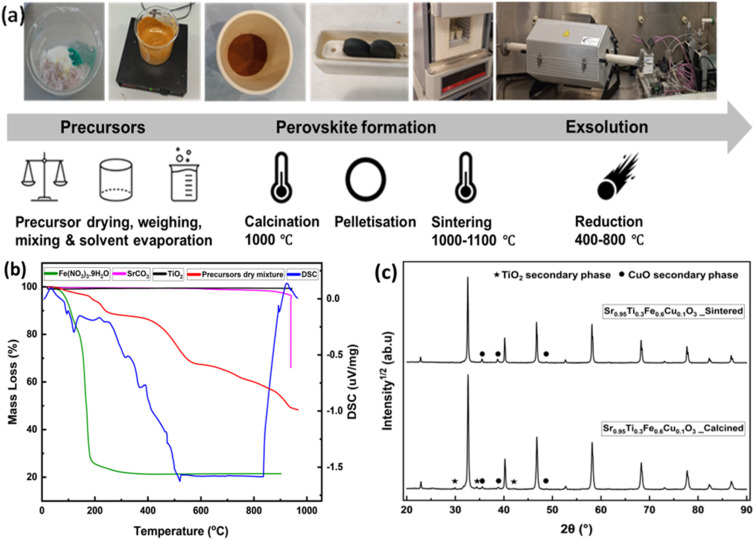
Perovskite synthesis process development and preliminary phase analysis results. (a) A schematic of the perovskite synthesis process; (b) a thermogram of Fe(NO_3_)_3_·9H_2_O (green), TiO_2_ (black) and SrCO_3_ (pink), the precursors' mixture dry powder (red), and the differential scanning calorimetry (DSC) curve (blue). (c) XRD peaks of Sr_0.95_Ti_0.3_Fe_0.6_Cu_0.1_O_3−*γ*_ showing improvement in phase purity after sintering.

The perovskites were synthesised using the solid-state synthesis method, with the key steps shown in Fig. 1(a). This involved calcinating dried precursor mixtures at 1000 °C, pellet formation and sintering in the air at 1000–1100 °C. The synthesis temperature was selected based on the thermal behaviour of the essential precursors—Fe(NO_3_)_3_·9H_2_O, SrCO_3_, and TiO_2_, as well as the dry mixture of these precursors, which was investigated using thermogravimetry analysis (TGA) and differential scanning calorimetry (DSC) (Fig. 1(b)).

Dehydration and nitrate decomposition resulted in a sharp mass loss for Fe(NO_3_)_3_·9H_2_O at 200 °C, whereas the observed mass loss around 800 °C corresponded to carbonate decomposition for SrCO_3_. The thermal analysis reveals a multi-step transformation process of the precursor material mixture up to 1000 °C, characterised by three main events: an initial dehydration around 200 °C, gradual decomposition between 300 °C and 600 °C, and a final crystallisation event at approximately 900 °C. The DSC curve and mass loss data indicate that the material undergoes complete transformation by 900 °C, resulting in a thermally stable product, which likely represents the formation of the incipient perovskite phase. Hence, 1000 °C was chosen as calcination temperature to ensure precursors' decomposition were complete and to promote the solid-state reaction necessary for full perovskite phase formation.

XRD analysis (Fig. 1(c)) revealed that the primary perovskite phase was successfully formed, accompanied by some secondary phases, including TiO_2_ and CuO, after calcination. However, sintering at 1000–1100 °C reduced the TiO_2_ secondary phase, resulting in a perovskite with a minimal CuO secondary phase and an improved structural composition.

### Crystal structure and composition of the Sr_0.95_Ti_0.3_Fe_0.7−*x*_Cu_x_O_3−*γ*_ systems

**Fig. 2:**
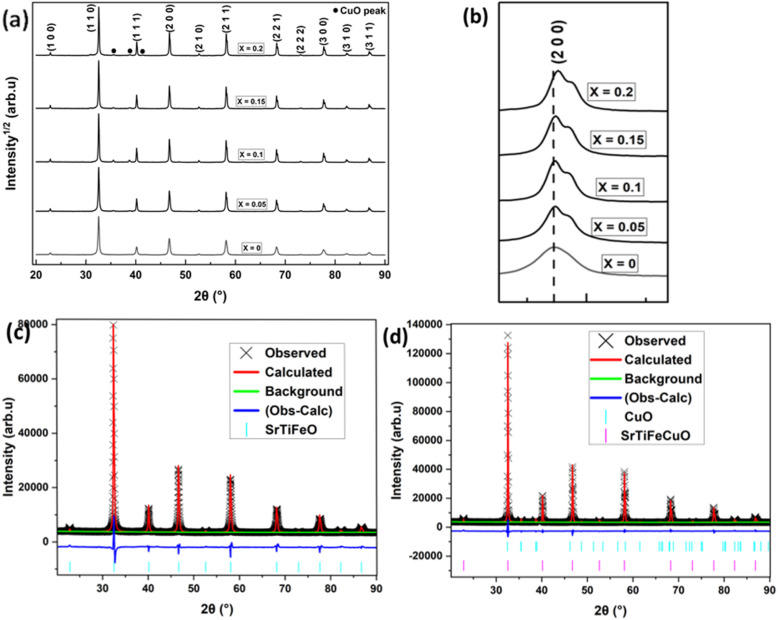
Phase analysis of the synthesised perovskite systems, Sr_0.95_Ti_0.3_Fe_0.7−*x*_Cu_*x*_O_3−*γ*_: (a) room temperature XRD pattern, highlighting the additional peaks resulting from doping (b) the (2 0 0) peaks showing the slight shift to a higher angle, with increasing *x*; (c and d) Rietveld refinement of sintered samples of (c) Sr_0.95_Ti_0.3_Fe_0.7_O_3−*γ*_ and (d) Sr_0.95_Ti_0.3_Fe_0.6_Cu_o.1_O_3−*γ*_, showing the primary phase, and the secondary phase, CuO.

The structural investigation revealed that all Sr_0.95_Ti_0.3_Fe_0.7−*x*_Cu_*x*_O_3−*γ*_ perovskite compositions exhibited a cubic structure (space group: *Pm*3̄*m*). This result (Fig. 2(a–d)) shows that the addition of Cu-doping up to *x* = 0.2 does not alter the cubic structure of Sr_0.95_Ti_0.3_Fe_0.7−*x*_Cu_*x*_O_3−*γ*_, despite the presence of a CuO secondary phase, indicating incomplete incorporation of the Cu dopant.

The Rietveld refinement of the sintered Sr_0.95_Ti_0.3_Fe_0.7−*x*_Cu_*x*_O_3−*γ*_ (*x*: 0–0.1) perovskite structure (Fig. 2(c and d) and Table 2) demonstrates good agreement between the observed and calculated patterns, confirming the successful synthesis of the target perovskite phase. The cell parameter ‘*a*’ shows a slight overall decreasing trend from 3.8860 Å to 3.8788 Å with increasing Cu content (*x*), while the crystallite size initially increases sharply from 0.11 µm in the undoped sample to ∼0.58–0.63 µm upon Cu doping but then remains relatively stable. Notably, the actual Cu incorporation (x*) is significantly lower than the nominal content at low doping levels. Still, it approaches the nominal value as the Cu content increases, suggesting a limit to Cu substitution in the structure at lower doping levels. The actual Cu-doping amount and resulting perovskites' stoichiometry (Table 2) were determined based on the CuO secondary phase weight fractions in the perovskite after synthesis, as explained in Note S1. The data show that forming the CuO secondary phase limited the Cu-doping incorporated into the perovskite structure, altering the designed stoichiometry and the envisaged A-site deficiency. Hence, we suspected the perovskite's exsolution capability would be negatively impacted. However, incorporating Cu into the perovskite lattice is expected to introduce additional oxygen vacancies, as reported for related perovskite structures.^26,27^ At the same time, Cu ions are among the most reducible in the first-row transition metals series (most negative Gibbs energy for the reduction), which would represent a significant driving force for exsolution to occur.^28,29^ Therefore, as a net result, the deviation from the intended A-site deficiency would likely not significantly limit the perovskite's exsolution capability.

**Table 2:** Structural and compositional parameters of Sr_0.95_Ti_0.3_Fe_0.7−*x*_Cu_*x*_O_3−*γ*_ systems (*x* = 0–0.2, space group: *Pm*3̄*m*) calculated from Rietveld refinement. Stoichiometries are reported in moles per mol of perovskite^a^

Nominal x* (mol)	0.00	0.05	0.1	0.15	0.2
Cell parameter *a* (Å, ± 5 × 10^−4^)	3.8870	3.8850	3.8820	3.8810	3.8780
Crystallite size (µm, ± × 10^−1^)	0.11	0.58	0.63	0.54	0.60
Amount of CuO (mol, ± × 10^−2^)	—	0.046	0.083	0.085	0.107
Actual *x*^a^ (mol, ± 5 × 10^−2^)	—	0.010	0.020	0.070	0.110
Perovskite stoichiometry	Sr_0.95_Ti_0.3_Fe_0.7_ O_3−*γ*_	SrTi_0.32_Fe_0.68_ Cu_0.01_O_3−*γ*_	Sr_1.04_Ti_0.33_Fe_0.65_ Cu_0.02_O_3−*γ*_	Sr_1.04_Ti_0.33_Fe_0.6_ Cu_0.07_O_3−*γ*_	Sr_1.06_Ti_0.34_Fe_0.56_ Cu_0.11_O_3−*γ*_
Goodness of fit (a.u.)	3.05	1.39	2.70	2.86	4.48

a Cu-doping amount.

### Microstructure of the Sr_0.95_Ti_0.3_Fe_0.7−*x*_Cu_x_O_3−*γ*_ systems

**Fig. 3:**
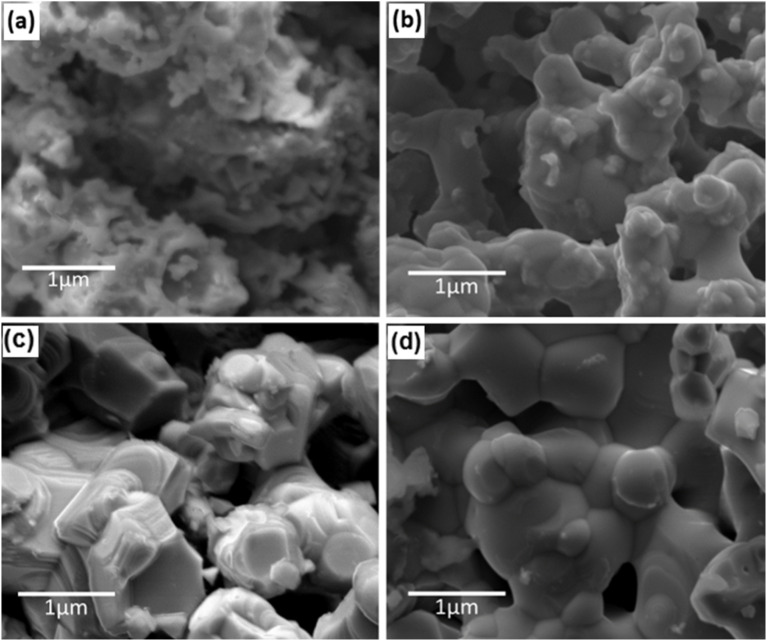
SEM image showing the microstructure of various perovskites. (a) Sr_0.95_Ti_0.3_Fe_0.7_O_3−*γ*_ (*x*: 0) after calcination at 1000 °C, (b) Sr_0.95_Ti_0.3_Fe_0.7_O_3−*γ*_ (*x*: 0) after sintering at 1000 °C and (c) Sr_0.95_Ti_0.3_Fe_0.6_Cu_0.1_O_3−*γ*_ (*x*: 0.1) after calcination at 1000 °C and (d) Sr_0.95_Ti_0.3_Fe_0.6_Cu_0.1_O_3−*γ*_ (*x*: 0.1) after sintering at 1000 °C.

The SEM analysis revealed well-formed grains with distinct grain boundaries and a better surface texture for the sintered Sr_0.95_Ti_0.3_Fe_0.7−*x*_Cu_*x*_O_3−*γ*_ (*x* = 0–0.2) perovskite compared to the calcined samples (Fig. 3(a–d)). The sintered sample of the Sr_0.95_Ti_0.3_Fe_0.7−*x*_Cu_*x*_O_3−*γ*_ (*x* = 0) perovskite contained interconnected grains with uniform porosity and grain sizes from ∼0.2 to 1.5 µm. On the other hand, sintered samples of the Cu-doped Sr_0.95_Ti_0.3_Fe_0.7−*x*_Cu_*x*_O_3−*γ*_ (*x* = 0.05–0.2) perovskite showed evidence of enhanced sintering and larger grain sizes between ∼0.5 and 3 µm. The observed microstructural evolution suggests that the sintering and Cu incorporation promote densification and enhance grain boundary development, resulting in significant grain growth.^30^

### Reduction kinetics of the Sr_0.95_Ti_0.3_Fe_0.7−*x*_Cu_*x*_O_3−*γ*_ perovskite system

**Fig. 4:**
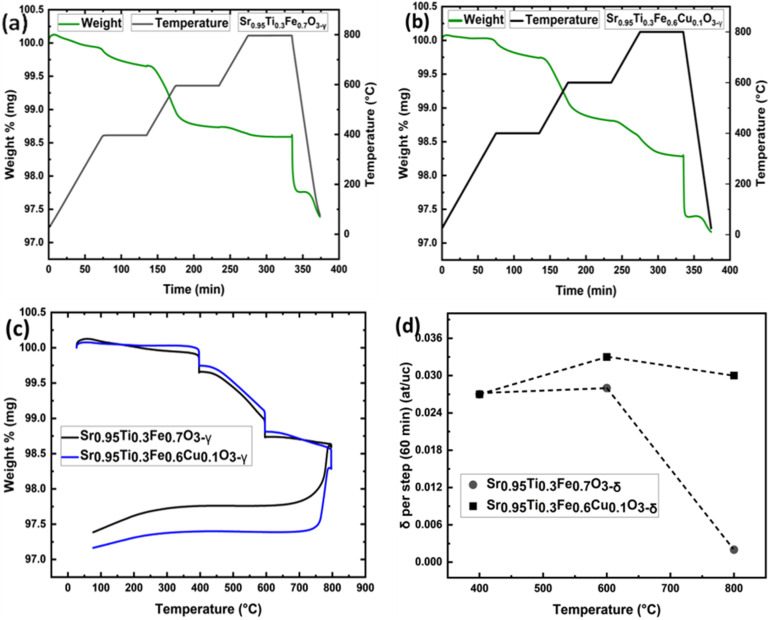
Thermograms and oxygen deficiency (*δ*) plots from the H_2_-TGA of Sr_0.95_Ti_0.3_Fe_0.7−*x*_Cu_*x*_O_3−*γ*_ (*x* = 0–0.1) perovskites showing mass loss and temperature *vs.* time plot of (a) Sr_0.95_Ti_0.3_Fe_0.7_O_3−*γ*_ and (b) Sr_0.95_Ti_0.3_Fe_0.6_Cu_0.1_O_3−*γ*_; (c) comparison of the plot weight loss *vs.* temperature for Sr_0.95_Ti_0.3_Fe_0.7_O_3−*γ*_ (black) and Sr_0.95_Ti_0.3_Fe_0.6_Cu_0.1_O_3−*γ*_ (blue); and (d) oxygen deficiency *vs.* temperature plot of Sr_0.95_Ti_0.3_Fe_0.7_O_3−*γ*_ and Sr_0.95_Ti_0.3_Fe_0.6_Cu_0.1_O_3−*γ*_ perovskite material. The straight, dotted line serves as a guide for the eye.

The exsolution of metallic nanoparticles from the perovskite lattice occurs through several coupled steps: removing oxygen ions to create vacancies, which simultaneously introduces electrons into the lattice, essential for reducing B-site cations (likely Cu), ultimately leading to their nucleation as metallic particles on the surface. Therefore, quantifying oxygen removal kinetics and the total extent of oxygen loss provides crucial insights into both the rate-limiting steps of the exsolution process and the amount of metal ions being reduced, enabling rational design of reduction conditions for controlled nanoparticle formation. The kinetics and extent of oxygen loss can be precisely monitored by tracking the material's weight changes during reduction using thermogravimetric analysis under hydrogen (H_2_-TGA), where controlled temperature steps allow us to systematically investigate the oxygen transport and reduction processes that underpin exsolution.^31^ The reduction kinetics of Sr_0.95_Ti_0.3_Fe_0.7−*x*_Cu_*x*_O_3−*γ*_ (*x* = 0 and 0.1) perovskite was investigated using thermogravimetric analysis under a hydrogen atmosphere (H_2_-TGA). The reduction mechanism was studied at three isothermal temperature steps: 400 °C, 600 °C, and 800 °C, with detailed comparisons of weight loss and oxygen deficiency (Fig. 4(a–d)).

At 400 °C, a weight loss of approximately 0.23% was observed for both Sr_0.95_Ti_0.3_Fe_0.7_O_3−*γ*_ and Sr_0.95_Ti_0.3_Fe_0.6_Cu_0.1_O_3−*γ*_. Table 3 summarises the H_2_-TGA result and the oxygen deficiencies for the Sr_0.95_Ti_0.3_Fe_0.7_O_3−*γ*_ and Sr_0.95_Ti_0.3_Fe_0.6_Cu_0.1_O_3−*γ*_ perovskite. The mass losses are attributed to the lattice oxygen loss accompanying the reduction of Fe^4+^ → Fe^3+^ and the exsolution of Cu, as well as the reduction of the CuO secondary phase to Cu metal. CuO with a reduction temperature as low as 180 °C,^31^ hence, is expected to reduce first. At 600 °C, both samples exhibited additional weight losses, with the Cu-doped perovskite showing a slightly higher loss (0.28% compared to 0.24% for the undoped system). This stage is associated with further oxygen loss and the exsolution of Cu nanoparticles from the Cu-doped sample. As shown in the next section, the Cu-doped sample showed more significant and well-dispersed nanoparticle formation at this stage, verified by SEM imaging.

**Table 3:** Weight losses and calculated oxygen deficiency (*δ*) from the H_2_-TGA of Sr_0.95_Ti_0.3_Fe_0.7_O_3−*γ*_ (*x* = 0 and *x* = 1) perovskite. Only oxygen deficiency calculated from mass loss recorded at the isothermal temperature step was considered, as proposed in the literature^32^

Temperature (°C)	Weight loss (%)		Cumulative weight loss (%)		Oxygen deficiency *δ* (at per uc)		Cumulative *δ* (at per uc)	
	*x* = 0	*x* = 0.1	*x* = 0	*x* = 0.1	*x* = 0	*x* = 0.1	*x* = 0	*x* = 0.1
400	0.23	0.23	0.23	0.23	0.026	0.027	0.026	0.027
600	0.24	0.28	0.47	0.51	0.028	0.032	0.054	0.059
800	0.02	0.26	0.49	0.77	0.002	0.030	0.056	0.089

The final temperature step at 800 °C resulted in a minimal weight loss of 0.02% for the undoped sample and an additional weight loss of 0.26% for the Cu-doped samples. This minimal change for the undoped sample suggests that its reduction process was nearly complete by 600 °C. In contrast, the weight loss at 800 °C indicates a possibility for additional exsolved nanoparticle growth in the Cu-doped perovskites. Cu-doped perovskite displayed a cumulative oxygen deficiency reaching 0.089 at per uc, *vs.* 0.056 for the undoped composition (Table 3), the highest observed value, indicating greater reducibility due to Cu doping, consistent with one of the premises of the work. At the same time, the limited increase in oxygen deficiency at lower temperatures for the Cu-doped samples compared to the undoped samples is consistent with the observation that not all Cu substitutes in the perovskite as intended. As corroborated in the literature,^32^ in this work, the reduction behaviour of the undoped and Cu-doped Sr_0.95_Ti_0.3_Fe_0.7−*x*_Cu_*x*_O_3−*γ*_ perovskites was modelled using a double exponential decay function, following eqn (1):

1 *m*(*t*) = *f*_0_ + *f*_1_ e^−*k*_1_*t*^ + *f*_2_ e^−*k*_2_*t*^

where *m*(*t*) is the perovskite's mass at time *t* during isothermal reduction; *f*_0_ the sample mass at the isothermal step; *f*_1_ and *f*_2_ represent the fractional contributions of the two distinct kinetic processes; *k*_1_ and *k*_2_ are the respective time constants (kinetic parameters) associated with each process. This reflects two parallel, rate-controlling processes: (i) surface exchange kinetics (fast, *k*_1_) and (ii) bulk oxygen diffusion (slow, *k*_2_).^32,33^

**Fig. 5:**
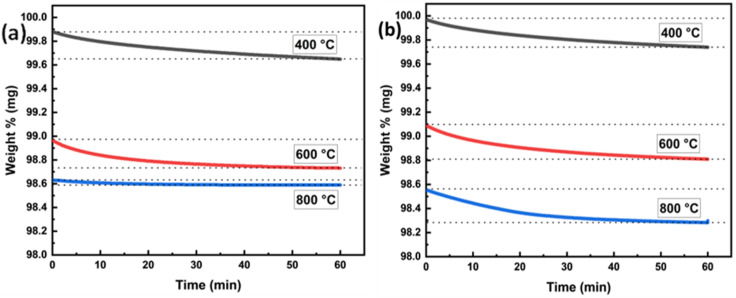
The reduction kinetics at the three isothermal temperature steps (400, 600, and 800 °C), based on mass loss due to structural oxygen loss from (a) Sr_0.95_Ti_0.3_Fe_0.7_O_3−*γ*_ and (b) Sr_0.95_Ti_0.3_Fe_0.6_Cu_0.1_O_3−*γ*_ perovskite. TGA was run in a 5% H_2_/95% Ar stream.

Fig. 5(a and b) compares the reduction kinetics of the undoped and Cu-doped systems. Our fitting revealed that Cu doping enhances the bulk diffusion component (*k*_2_), consistent with a modification of the oxygen vacancy energy landscape.

Table 4 presents the calculated parameters for the two perovskites at different isothermal temperature steps. For the perovskites studied here, the kinetic parameter, *k*_1_, had the highest values at 400 °C, the early stage of the reduction process.

**Table 4:** Fitting parameters for the reduction kinetics model of Sr_0.95_Ti_0.3_Fe_0.7_O_3−*γ*_ and Sr_0.95_Ti_0.3_Fe_0.6_Cu_0.1_O_3−*γ*_ perovskite materials

Fitting parameters	Perovskites at different temperatures					
	Sr_0.95_Ti_0.3_Fe_0.7_O_3−*γ*_			Sr_0.95_Ti_0.3_Fe_0.6_Cu_0.1_O_3−*γ*_		
	400 °C	600 °C	800 °C	400 °C	600 °C	800 °C
*f*_0_ (mg)	99.54	98.72	98.59	99.66	98.77	98.27
*f*_1_ (mg)	0.05	0.08	0.02	0.05	0.24	0.15
*K*_1_ (min^−1^)	0.15	0.19	0.08	0.14	0.03	0.06
*f*_2_ (mg)	0.29	0.17	0.02	0.26	0.08	0.15
*K*_2_ (min^−1^)	0.02	0.04	0.08	0.019	0.17	0.06

A striking feature in the Cu-doped sample is the dramatic shift in the *k*_2_/*k*_1_ ratio from 0.14 at 400 °C to 5.67 at 600 °C, before returning to 1.0 at 800 °C. This shift reveals a complex evolution of rate-determining steps with temperature. This non-monotonic behaviour suggests that different reduction mechanisms dominate at various temperature regimes, with bulk diffusion becoming particularly enhanced at intermediate temperatures. Nanoparticle exsolution and its impact on the bulk crystal and surface structure could also be contributing factors. At 600 °C, Cu-doping appears to fundamentally alter the reduction kinetics, significantly accelerating bulk processes (*k*_2_ = 0.17) while simultaneously slowing surface processes (*k*_1_ = 0.03) compared to the undoped sample (*k*_2_ = 0.04, *k*_1_ = 0.19). This inverse effect on surface *versus* bulk kinetics suggests that Cu-doping may modify oxygen transport pathways by affecting the material's vacancy formation and migration energies. However, it could also be related to competition with reactions such as exsolution, which has surface and bulk components.

The undoped sample shows an unusual evolution in the relative contributions of surface and bulk processes, with the *f*_1_/*f*_2_ ratio shifting from ∼0.17 at 400 °C to 1.0 at 800 °C. This indicates the initial dominance of bulk oxygen transport that eventually equilibrates with surface processes. In contrast, the Cu-doped sample maintains consistently higher *f*_2_ values throughout the temperature range, suggesting that Cu-doping promotes sustained bulk-mediated reduction processes even at higher temperatures. This result indicates that Cu-doping enhances the reducibility and bulk lattice oxygen transport capability of Sr_0.95_Ti_0.3_Fe_0.6_Cu_0.1_O_3−*γ*_, suggesting enhanced exsolution capability.

### Exsolution capability of the Sr_0.95_Ti_0.3_Fe_0.7−*x*_Cu_*x*_O_3−*γ*_ perovskite system

**Fig. 6:**
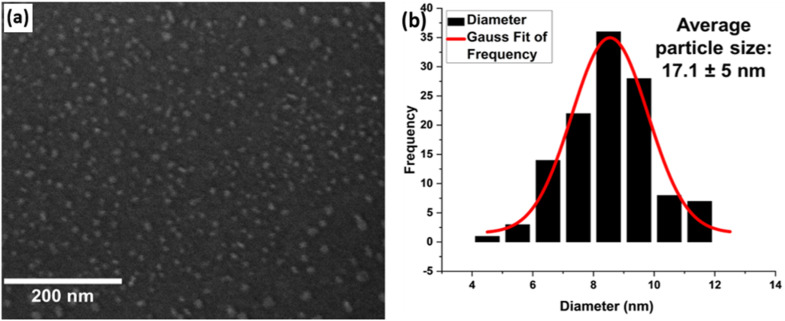
Micrograph image and the corresponding particle size analysis for Sr_0.95_Ti_0.3_Fe_0.7_O_3−*γ*_ (*x* = 0) perovskite, showing the structure following reduction at 600 °C. (a) SEM of observed exsolved nanoparticles, (b) corresponding particle size distribution.

**Fig. 7:**
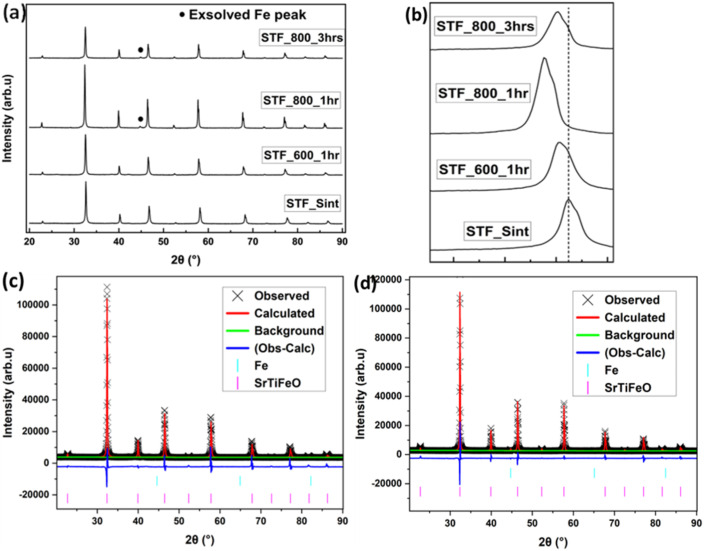
Phase analysis of the Sr_0.95_Ti_0.3_Fe_0.7−_O_3−*γ*_ perovskite system reduced at different temperatures. (a) Room temperature XRD pattern, highlighting the exsolved Fe peak clearly observed after reduction at 800 °C; (b) the (2 0 0) peaks showing the slight shift to a lower angle; (c and d) Rietveld refinement of the samples reduced at 600 °C and 800 °C: (c) sample reduced at 600 °C in 1 hour and (d) sample reduced at 800 °C in 1 hour, confirming Fe exsolution.

The exsolution behaviour of the Sr_0.95_Ti_0.3_Fe_0.7−*x*_Cu_*x*_O_3−*γ*_ perovskite system (*x*: 0–0.1) was investigated, focusing on the influence of Cu-doping, reduction temperature, and time on the material's structure and surface properties. Samples were reduced in 5% H_2_ at temperatures of 400, 600, and 800 °C and for durations ranging from 1 to 4 hours. Fig. 6(a and b) shows the SEM image and particle size analysis result of the undoped perovskite, Sr_0.95_Ti_0.3_Fe_0.7_O_3−*γ*_, reduced at 600 °C in 1 hour. Under these conditions, the observed particles display an average size of ∼17 nm. XRD and XPS analysis (Fig. 7(a–d) and later discussion) revealed important distinctions about particle formation in the undoped perovskite at different temperatures. At 400 °C, no particle formation occurred (Fig. S1). At 600 °C, while particles were observed (Fig. 6(a)), our data indicate these are iron oxide nanoparticles formed by segregation rather than true metallic iron exsolution. Genuine Fe metal nanoparticle exsolution only occurred at the higher temperature of 800 °C (Fig. 7(a–d)).

At 400 °C, this likely indicates an insufficient driving force for Fe exsolution from the undoped material. At 800 °C, the exsolution may have been suppressed, possibly due to surface restructuring or A-site enrichment, which is known to impact exsolution in perovskites.^34–36^ The oxygen loss measured at 600 °C correlates with particle formation observed in SEM images. However, XRD and XPS analyses (detailed later) confirm that iron remains in an oxidized state rather than forming metallic nanoparticles. This indicates that while the undoped perovskite undergoes structural changes and forms oxide particles at moderate temperatures, it cannot achieve true metallic exsolution below 800 °C. This temperature limitation restricts the material's usefulness for applications requiring low-temperature processing.

**Fig. 8:**
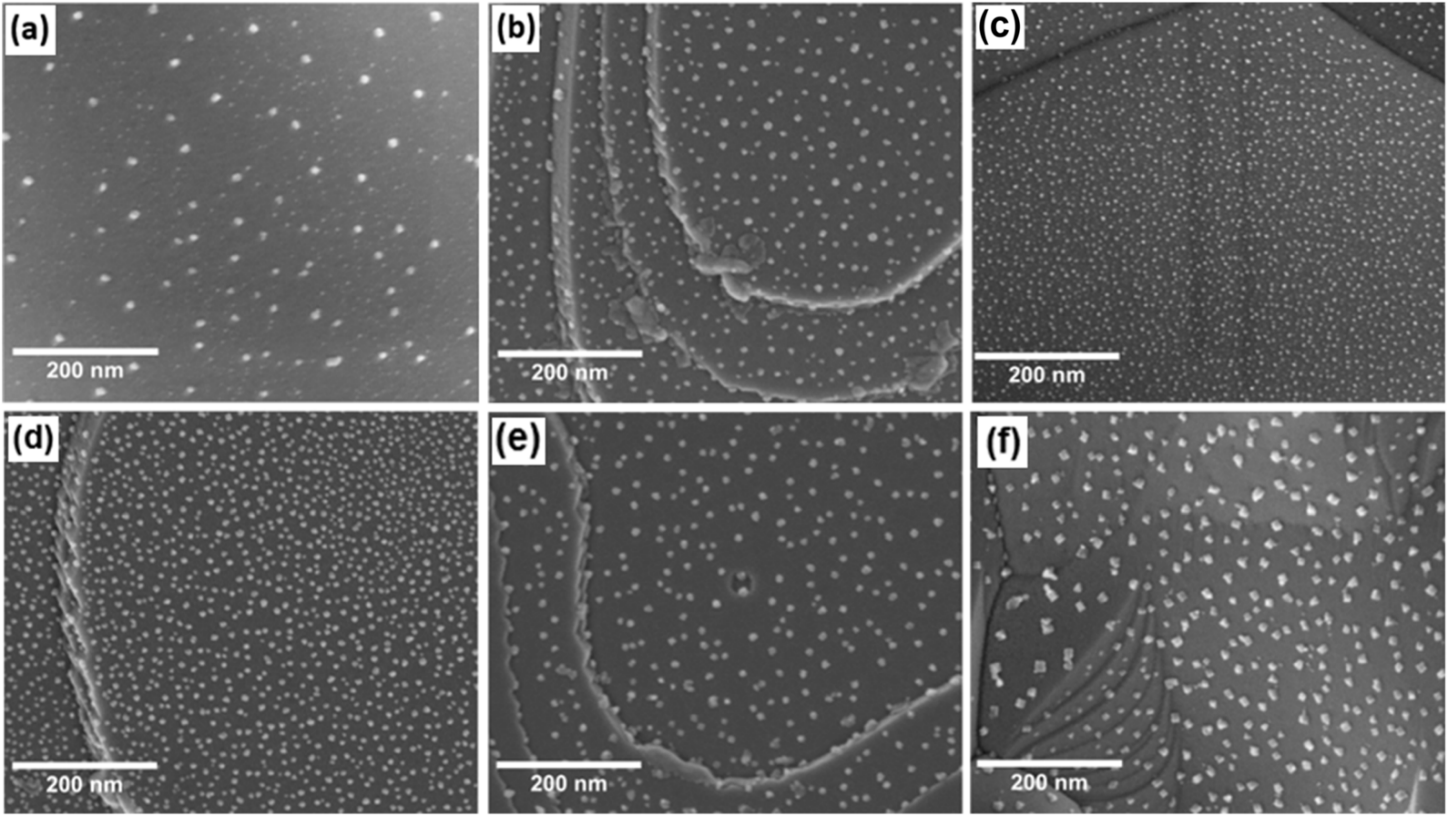
SEM micrograph images of Sr_0.95_Ti_0.3_Fe_0.6_Cu_0.1_O_3−*γ*_ perovskites after reduction at various temperatures, corresponding to observed exsolution at (a) 400 °C in 1 hour; (b–e) 600 °C in reduction times of 1, 2, 3, and 4 hours, respectively; and (f) 800 °C in 1 hour.

**Fig. 9:**
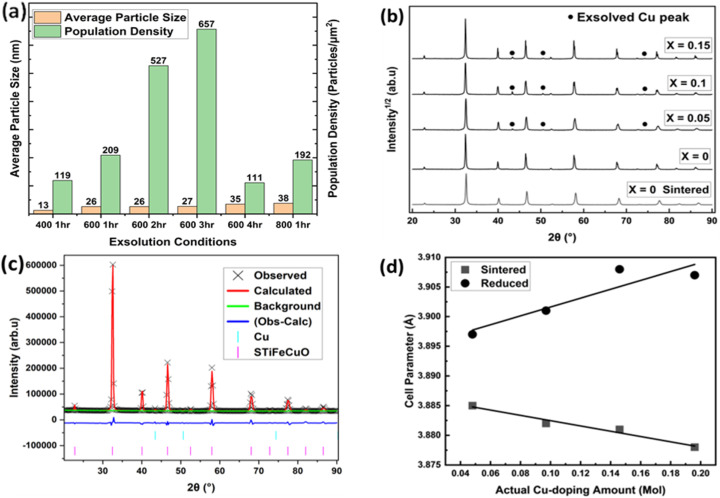
Particle size analysis result of Sr_0.95_Ti_0.3_Fe_0.6_Cu_0.1_O_3−*γ*_ perovskite under different reduction conditions; and XRD and Rietveld refinement result of the Sr_0.95_Ti_0.3_Fe_0.7−*x*_Cu_*x*_O_3−*γ*_ perovskite system reduced at 600 °C in 1 hour. (a) Particle sizes and population density under different reduction conditions (400 °C, not fully represented as a bimodal distribution on the plot); (b and c) XRD patterns: (b) showing peaks of Cu nanoparticles after reduction, (c) Rietveld refinement profile identifying the exsolved nanoparticles as metallic Cu, and (d) variation of perovskites' cell parameter with actual Cu-doping amount for the as-synthesized and reduced perovskites.

Fig. 8(a–f) show the SEM image while Fig. 9(a) shows the particle size analysis results for Sr_0.95_Ti_0.3_Fe_0.6−*x*_Cu_0.1_O_3−*γ*_ perovskite under various reduction conditions. See Fig. S1(d) for an SEM image of the Sr_0.95_Ti_0.3_Fe_0.6_Cu_0.1_O_3−*γ*_ pristine sample. This perovskite exhibited pronounced nanoparticle exsolution, particularly at temperatures of 600 °C and 800 °C. At 400 °C, relatively spherical nanoparticles were observed, with a bimodal distribution centered at approximately 13 and 24 nm, suggesting that these temperature and time conditions may be insufficient for achieving size equilibration.

Increasing the temperature to 800 °C resulted in multifaceted particle shapes with an average diameter of ∼38 nm. The influence of the reduction time was also investigated, specifically at 600 °C, where nanoparticle density increased from 207 ± 2 particles per µm^2^ at 1 hour to 650 ± 7 particles per µm^2^ at 3 hours. At 800 °C, the elevated temperature promoted further growth and morphological evolution of the nanoparticles, resulting in a decrease in the nanoparticle population to approximately 188 ± 4 particles per µm^2^, compared to the 207 ± 2 particles per µm^2^ obtained at 600 °C in 1 hour. The quoted error in the population density was determined, as explained in Note S2. See Table S1 for the summary of particle size analysis for Sr_0.95_Ti_0.3_Fe_0.7_O_3−*γ*_ and Sr_0.95_Ti_0.3_Fe_0.6_Cu_0.1_O_3−*γ*_.

XRD and Rietveld refinement (Fig. 9(b and c)) indicated successful exsolution driven by the incorporated Cu-dopant in the perovskite system. See Fig. S2 and Table S2 for the pattern and summary of the SEM-EDS result and the determined stoichiometry of the Sr_0.95_Ti_0.3_Fe_0.6_Cu_0.1_O_3−*γ*_ perovskite's surface composition.

The structural stability of the perovskite remained largely unaffected by the exsolution process, with only some changes in lattice parameters, as confirmed by the Rietveld refinement result. Fig. 9(d) shows that the cell parameters decreased with the Cu-doping amount for the sintered samples (3.886–3.878 Å) and increased for the reduced samples, reaching a maximum of 3.908 Å. See Table S3 for the cell parameters of the perovskites derived from the Rietveld refinement. The increase in lattice parameters was attributed to the reduction of Fe^4+^ to Fe^3+^ (which increases the average B-site cation size), oxygen vacancy formation, and the exsolution of Cu nanoparticles, which contributed to charge compensation *via* the removal of Cu^2+^ cations from the lattice.

The findings presented here demonstrate that the Sr_0.95_Ti_0.3_Fe_0.7−*x*_Cu_*x*_O_3−*γ*_ perovskite system, particularly with Cu doping, despite limited substitutions, has strong potential for the controlled exsolution of nanoparticles, making it a promising candidate for catalysis applications.

### Chemical state characterization of exsolved perovskites by XAS and surface characterisation by XPS

**Fig. 10:**
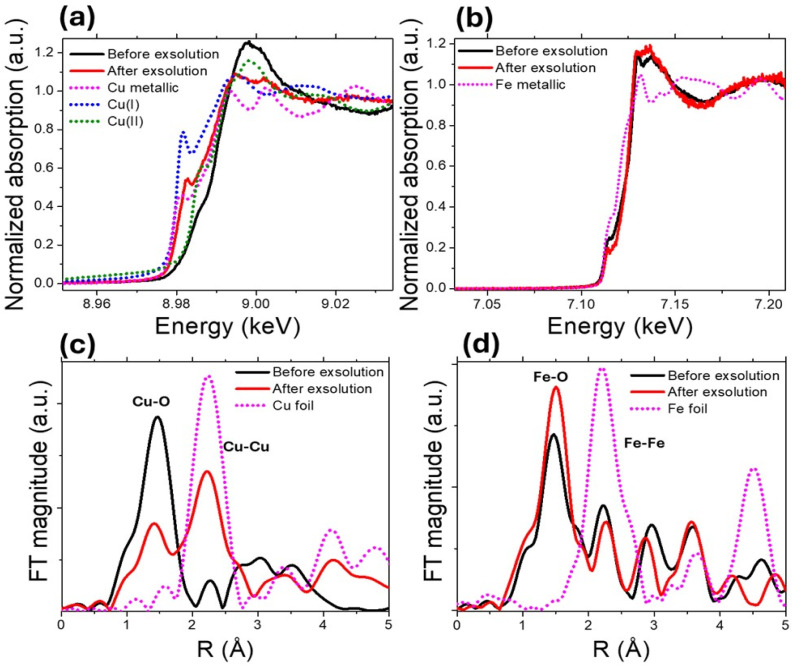
*In situ* Cu and Fe K-edge XAS spectra of the Cu-doped perovskite collected before (black) and after (red) exsolution at 600 °C for 1 h. (a) Cu K-edge XANES spectrum shows a shift to lower energies and a reduced white-line intensity, indicating partial reduction of Cu species and the emergence of Cu(i)/Cu^0^ features after exsolution. (b) Fe K-edge XANES displaying only subtle spectral changes, suggesting that Fe remains largely unaffected during reduction. (c) Fourier-transformed EXAFS magnitude plot showing that the Cu–Cu peak markedly increases after reduction, confirming metallic Cu nanoparticle formation. (d) Fourier-transformed EXAFS magnitude plot showing that Fe–O and Fe–Fe contributions show minimal change, indicating limited structural modification of Fe.

To understand how the thermal treatment affects the oxidation state of copper and iron, we performed *in situ* XAS measurements in the Cu and Fe edges before and after the exsolution process at 600 °C for 1 h (Fig. 10(a and b)). Before exsolution (black curve), the X-ray Near Edge Structure (XANES) profile exhibits an elevated white line characteristic of Cu oxides,^37^ in line with all the previous characterization. After exsolution (red curve), the absorption edge shifts to lower energy values, accompanied by a reduction in white-line intensity, suggesting a reduction of Cu species. The spectrum becomes more similar to that of Cu(i) or metallic Cu (pink curve), again in line with the previous characterization (XRD) showing the presence of metallic Cu. The linear combination analysis yielded fractions of 0.28 ± 0.02 for Cu^0^, 0.50 ± 0.02 for Cu^1+^, and 0.22 ± 0.04 for Cu^2+^ after exsolution, with an R-factor of 0.0018. Therefore, there remains a considerable amount of Cu^1+^ after exsolution, which explains why its XANES profile considerably differs from that of metallic Cu.

On the other hand, the changes are much less clear in the case of the Fe edge, indicating that Fe atoms undergo some relatively minor changes in comparison with Cu, confirming that the exsolution occurs mainly due to the reduction of Cu, and then the consequent formation of pure metallic Cu nanoparticles. We cannot discard the possibility of some alloying but, if it is the case, the XAS experiments show that the nanoparticles should be highly enriched in Cu.

The Extended X-ray Absorption Fine Structure (EXAFS) analysis (Fig. 10(c and d)) reinforces what is observed with XANES. After exsolution, there is a remarkable increase in the Cu–Cu bond peak, while the Fe–Fe peak barely changes.

**Fig. 11:**
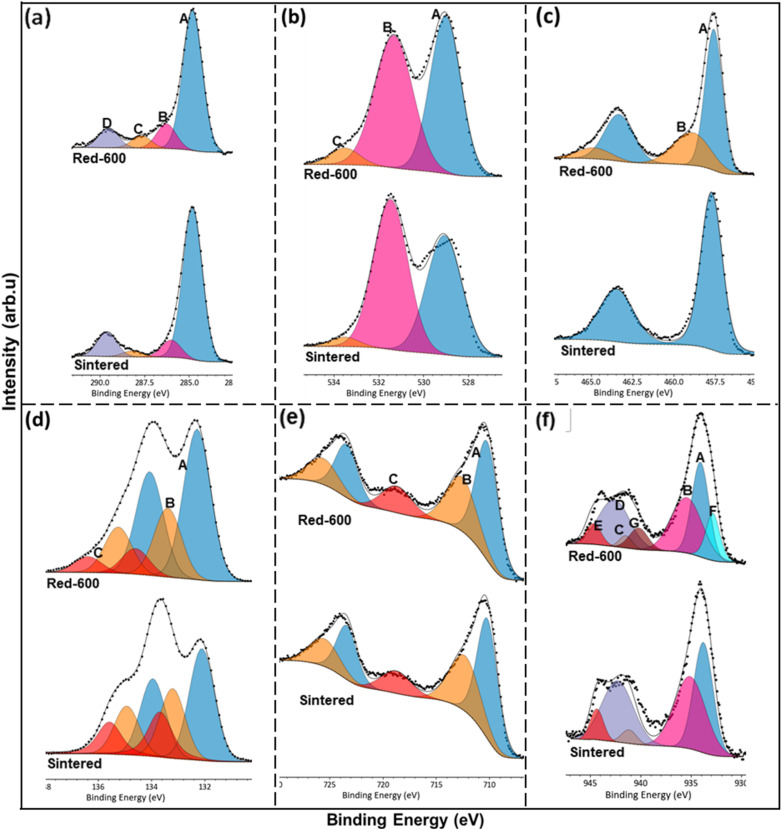
Surface analysis by XPS for Sr_0.95_Ti_0.3_Fe_0.7−*x*_Cu_*x*_O_3−*γ*_ perovskites. Core spectra of C 1s, O 1s, Ti 2p, Sr 3d, Fe 2p and Cu 2p, for the sintered and reduced (600 °C, 1 h) sample, after deconvolution. (a) C 1s spectra deconvoluted into four components [A = C–C, B = C–O, C = C=C; and D = O–C=O]; (b) O 1s spectra deconvoluted into 3 components [A = O-Lat, B = O-surface, and C = O–CO/O–OH]; (c) Ti 2p deconvoluted into 1 component [A = Ti^4+^ doublet] for both the sintered and reduced sample, and two additional satellite peaks [B = Ti^4+^ satellite peaks] for the reduced sample; (d) Sr 3d spectra deconvoluted into 3 components [A = Sr-Lat doublet, B = SrCO_3_ doublet, and C = SrO_(1−*X*)_ doublet]; (e) Fe 2p spectra deconvoluted into 2 components and a satellite peak [A = Fe^3+^ doublet, B = Fe^4+^ doublet, and C = Fe^3+^ satellite peak]; and (f) Cu 2p, deconvoluted into 2 components and 3 satellite peaks for the sintered and reduced sample [A = Cu^2+^, B = Cu^2+^ isolated atom, C–E = satellite peaks 1, 2, and 3], plus additional 1 component and 1 satellite peak [ F = Cu^0^, and G = Cu^0^ satellite peak] for the reduced sample. The different species of all the fitted spectra are summarised in Tables S4 and S9.

**Fig. 12:**
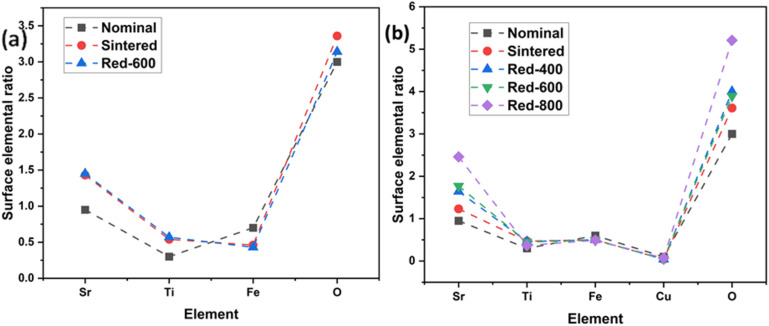
Surface stoichiometry by XPS of the Sr_0.95_Ti_0.3_Fe_0.7−*x*_Cu_*x*_O_3−*γ*_ perovskite system showing the deviation of different processed samples from the nominal (as-designed) sample. (a) Comparison of the Sr_0.95_Ti_0.3_Fe_0.7_O_3−*γ*_ perovskite sintered and reduced samples with the nominal sample. (b) Comparison of the Sr_0.95_Ti_0.3_Fe_0.7−*x*_Cu_*x*_O_3−*γ*_ perovskite sintered and reduced (400, 600, and 800 °C) samples with the nominal sample.

Furthermore, the XPS core-level spectra (Fig. 11, S3 and S4) collected on both the undoped STF and Cu-STF materials in their as-sintered state and after H_2_ reduction (400–800 °C) provide supporting information that confirms metallic Cu as the only exsolved metallic component at 600 °C. Fig. 11 Compares the high-resolution core spectra of C 1s, O 1s, Ti 2p, Sr 3d, Fe 2p and Cu 2p, for Sr_0.95_Ti_0.3_Fe_0.7−*x*_Cu_*x*_O_3−*γ*_ sintered and reduced (600 °C, 1 h) sample, after deconvolution. The XPS analysis shows that Sr_0.95_Ti_0.3_Fe_0.7−*x*_Cu_*x*_O_3−*γ*_ surfaces are Sr-enriched with mixed Fe^3+^/Fe^4+^ states and surface oxygen overstoichiometry. Cu doping increases surface oxygen and carbonate adsorption, while reduction drives oxygen vacancy formation, and Cu exsolution. The Cu 2p spectra reveal a mixed Cu^2+^/Cu^0^ environment under reducing conditions, with Cu^2+^ sites believed to aid nitrate adsorption while the Cu^0^ nanoparticles enhance the reduction. Overall, both Sr_0.95_Ti_0.3_Fe_0.7_O_3−*γ*_ and Sr_0.95_Ti_0.3_Fe_0.6_Cu_0.1_O_3−*γ*_ surfaces deviate significantly from bulk stoichiometry, with these changes intensifying upon reduction (Fig. 12(a and b) and Table S10 as well as Fig. S5).

XPS analysis reveals notable deviations of surface composition from the nominal stoichiometry according to XRD Rietveld refinement, as shown in Table S10, for both Sr_0.95_Ti_0.3_Fe_0.7_O_3−*γ*_ and Sr_0.95_Ti_0.3_Fe_0.6_Cu_0.1_O_3−*γ*_. In all cases, Sr is enriched at the surface, increasing with reduction temperature, particularly in the Cu-doped perovskite (showing a Sr/B site ratio of 1.63 → 2.46), indicating pronounced A-site migration under reducing conditions. Fe ratio is low relative to nominal, for example: decreasing from 0.7 in the nominal to 0.43 in the undoped reduced sample. On the other hand, Ti remains higher than the nominal, indicating Sr segregation driven surface reconstruction. The Fe^3+^ to Fe^4+^ ratio increases with reduction, confirming partial Fe^4+^ → Fe^3+^ reduction and corresponding oxygen vacancy formation. Cu ratio remains lower than the nominal value under all conditions, though increasing with reduction temperature, indicating the increased extent of exsolution at elevated temperatures. Surface oxygen ratios remain above 3, consistent with oxygen over-stoichiometry arising from Sr segregation and CO_3_ and oxide species adsorption.

Across processing conditions, the evolution of Sr enrichment, Fe depletion, and Fe redox transitions reflects a dynamic balance between reduction and cation redistribution. Moderate reduction (400–600 °C) provides the most favourable surface state for catalytic redox activity, characterised by accessible Fe^3+^/Fe^4+^ or Cu^0^ sites and oxygen vacancies, and moderate Sr segregation. The excessive Sr segregation and oxygen over-stoichiometry at higher temperatures (*e.g.*, Red-800) likely produce insulating Sr-rich surface layers that hinder charge transfer and block active sites. These findings reiterate that Cu incorporation enhances surface stability and redox flexibility, while controlled reduction is essential for maximising catalytic performance and maintaining structural integrity.

### Electrochemical characterization

To analyse the surface features of Sr_0.95_Ti_0.3_Fe_0.6_Cu_0.1_O_3−*γ*_ before and after exsolution at different temperatures, we conducted a reduction treatment (5% H_2_ and 95% Ar) at 200 °C (where no exsolution is evident) and at intermediate temperatures of 400 °C and 600 °C for 1 hour. The 200 °C treatment was added to reduce the gap (and then have a smoother transition) between the reduced and non-reduced samples.

**Fig. 13:**
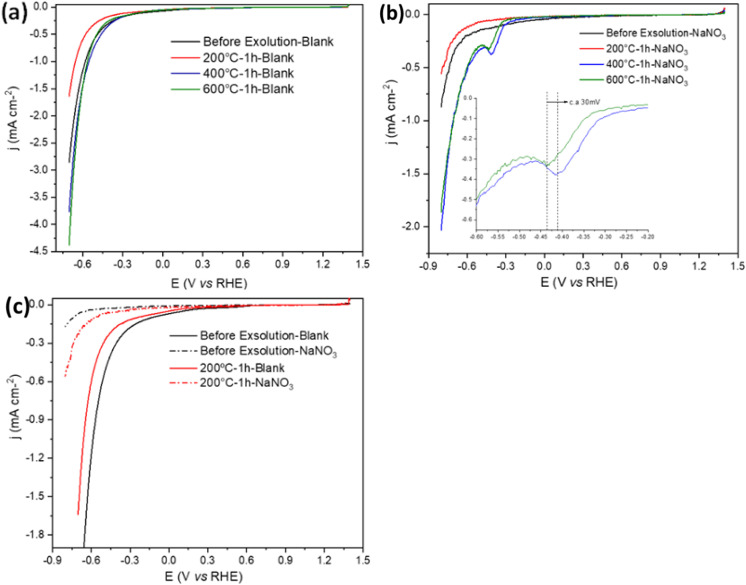
The cathodic sweep of CVs for Sr_0.95_Ti_0.3_Fe_0.6_Cu_0.1_O_3−*γ*_ before exsolution and after exsolution at 200 °C, 400 °C, and 600 °C for 1 h in (a) 0.1 M NaOH; (b) in 0.1 M NaOH and 1 mM NaNO_3_ at 50 mV s^−1^ (inset: NO_3_RR peaks for 400 °C and 600 °C samples); (c) comparison of Sr_0.95_Ti_0.3_Fe_0.6_Cu_0.1_O_3−*γ*_ before exsolution and exsolved at 200 °C for 1 h in 0.1 M NaOH *vs.* 0.1 M NaOH with 1 mM NaNO_3_. All the potentials are referred to RHE.

Even if electrochemical measurements are most commonly used to test the activity and stability of materials, these can be extremely useful for characterising some surfaces.^38^ Thus, in this work, we have utilised the interaction between nitrate ions and Cu to monitor changes at the surface of Cu-based materials qualitatively. Fig. 13(a and b) shows the cathodic sweep of cyclic voltammograms (CVs) recorded in the absence and presence of NaNO_3_, respectively.

The choice of nitrate provides insights into the NO_3_RR (electro)catalytic activity changes before and after exsolution, serving as a surface probe for material characterisation.^39^ Shifts in the reduction potentials or current densities indicate changes in surface chemistry.^40^ In the absence of nitrate, notable reduction currents were observed for all samples at more negative potentials than −0.3 V_RHE_ (Fig. 13(a)), corresponding to the hydrogen evolution reaction (HER) and which is typical of metallic and metallic oxide surfaces.^41–43^

However, it is worth noting that the sample treated at 200 °C showed notably lower HER activity. We argue that the CuO secondary phase was reduced (as discussed earlier) to a less active Cu fraction, which lacks the enhanced catalytic properties of Cu nanoparticles or the CuO phase existing in the sample before exsolution. This change likely results from the altered distribution of Cu oxidation states and surface oxidation coupled with complex electrochemical conditions. Identifying active sites and understanding the interplay of various parameters influenced by thermal treatment or exposure to the electrochemical environment—such as surface oxidation, oxophilicity, and hydrophilicity—remain challenging.^30^ Nonetheless, these results highlight the sensitivity of electrochemical methods as powerful tools for characterising the surface properties of such materials.^41^

For the samples reduced at 400 and 600 °C, in the presence of NO_3_^−^, a peak emerges between −0.3 and −0.4 V_RHE_ due to the NO_3_RR.^44^ A steep increase follows this in the current reduction due to the HER. The hydrogen coverage on the surface of these Cu-based catalysts increases as the potential is scanned in the negative direction. However, while hydrogen adsorption is essential for forming products such as ammonia, excessive hydrogen coverage can block active sites (likely the Cu nanoparticles), thus inhibiting further NO_3_RR. This blockage eventually leads to the re-emergence of the HER.^45^Fig. 13(b) shows differences for the sample exsolved at 400 and 600 °C. The latter exhibits a lower current peak intensity and a shift of approximately 30 mV towards a more negative potential. The lower intensity is likely due to the larger size of the exsolved nanoparticles at this temperature (as shown in the SEM images in Fig. 8(b)), resulting in a lower surface area and reduced NO_3_RR activity. Still, changes in the composition, either of the nanoparticles or in the support near the nanoparticles, cannot be discarded.

Finally, for the samples before exsolution or those reduced at 200 °C for 1 hour (Fig. 13(c)), we did not observe the characteristic peak of the NO_3_RR. These materials are inert for this reaction under these conditions, and the currents observed follow the same trend as in the absence of NO_3_^−^, suggesting that in both cases, we are observing the response of the HER. However, a shift toward a more negative HER onset potential when NO_3_^−^ ions are present indicates the presence of Cu species on the surface,^46^ possibly in the form of Cu ions within the perovskite lattice surface and/or CuO secondary phase.

Furthermore, while the Sr_0.95_Ti_0.3_Fe_0.7_O_3−*γ*_ support would provide some level of mixed conductivity and oxygen-vacancy sites as earlier discussed, according to Fig. 10(a–c), it shows no intrinsic NO_3_RR activity in the absence of exsolved Cu (onset only at −0.20 V RHE, no NO_3_RR peak). Only upon Cu nanoparticle exsolution (400–600 °C) does a clear NO_3_RR signal arise (Fig. 10(b)), with the most positive onset and highest currents seen for the finest particles (400 °C). Thus, although support interactions may modulate selectivity, the predominant enhancement in nitrate reduction performance is governed by the presence and size of the exsolved Cu^0^ nanoparticles. Importantly, the electrochemical results are used here primarily as a proof-of-concept to demonstrate how controlled exsolution can be used to tune material properties rather than to optimize performance for nitrate reduction specifically.

## Experimental

### Material synthesis

An A-site deficient Sr_0.95_Ti_0.3_Fe_0.7−*x*_Cu_*x*_O_3−*γ*_ perovskite system with varied Cu-doping (*x* = 0, 0.05, 0.1, 0.15, 0.2 mol) was designed and synthesised using the solid-state synthesis method. The following precursors were used: SrCO_3_ (99.99% purity, Thermo Fisher), TiO_2_ (99.5% purity, Sigma-Aldrich), Fe(NO_3_)_3_·9H_2_O (98%, Alfa Aesar), and Cu(NO_3_)_2_·2.5H_2_O (98% purity, Thermo Fisher).

The solid-state synthesis (Fig. 1(a)) involved calcining dried precursor mixtures at 1000 °C, pellet formation and sintering in air at 1000–1100 °C. The synthesis temperature was selected based on the thermal behaviour of essential precursors—Fe(NO_3_)_3_·9H_2_O, SrCO_3_, and TiO_2_, as well as the dry mixture of these precursors, using thermogravimetry analysis (TGA) and differential scanning calorimetry (DSC). Netzsch STA 449F3 equipment was used for the TGA. The TGA was run at 30–950 °C for Fe(NO_2_)_3_·9H_2_O, SrCO_3_ and TiO_2_ at a 10 °C min^−1^ heating rate. Subsequently, the TGA was run at 30–1010 °C for the dried mixture of the precursors before calcination to Sr_0.95_Ti_0.3_Fe_0.7_O_3−*γ*_.

### Actual perovskite stoichiometry determination

The actual Cu-doping amount, *y*, and the resulting perovskite stoichiometry were determined using eqn (2) (eqn (S3) in the SI).2
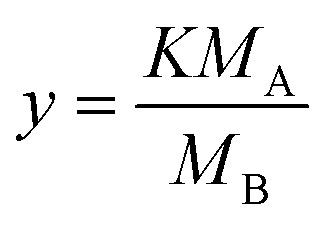


The derivation and parameters of eqn (S3) are explained in Note S1.

### Reduction of the sample

Reduction samples were prepared by breaking the sintered pellets into small pieces to have samples from the same pellet for different reduction temperatures and times. This ensured that any observed variation was not associated with factors other than the reduction parameters and the material's intrinsic properties. The prepared samples were reduced at different temperatures (400, 600, and 800 °C) in 5% H_2_/Ar (95%). All reduction process was done at the same reduction time of 1 hour. To investigate the effect of reduction time, samples were reduced at 600 °C for 1, 2, 3, and 4 hours respectively. All reduction was initiated at a room temperature of 25 °C, with a ramp rate of 5 °C min^−1^.

### Crystal structure study

X-ray diffraction (XRD) analysis was employed to investigate the calcined and sintered perovskite crystal structure. A Rigaku equipment with Cu-Kα radiation (*λ*: 1.5406 Å) was utilised. The scanning was performed with a step size of 0.02°, a scan rate of 3° min^−1^, and a scanning range of 20–90°. The Rietveld refinement of the synthesised and reduced perovskites was done using the GSAS II software. The reference structures used for the refinement were Ti_0.5_Fe_0.5_SrO_0.29_ (1525550)^53^ and CuO (1011148)^54^ for the as-sintered perovskite, while Ti_0.5_Fe_0.5_SrO_0.29_ (1525550) and Cu (4105681)^55^ were used for the reduced perovskites. The unavailability of A-site deficient (Sr_1−*x*_Ti_*y*_Fe_1−*y*_O_3−*γ*_) perovskite's CIF with Cu-doping on the B-site warranted the choice of the Ti_0.5_Fe_0.5_SrO_0.29_ CIF. Also, the phase analysis on the Rigaku Miniflex suggested that the chosen structures could be phase matches for the respective perovskites.

### Microstructure study

Scanning electron microscopy (SEM) was used to study the microstructure of the calcined and sintered perovskite samples. All analyses were performed at the Geoanalytical Electron Microscopy and Spectroscopy (GEMS) facility within the School of Geographical and Earth Sciences at the University of Glasgow using Zeiss Sigma VP FEG SEM equipment. Furthermore, SEM analysis was also employed to investigate the reduced perovskites' microstructure and the exsolved nanoparticles' morphology. Sample imaging was performed using a secondary electron and in-lens detector, with an accelerating voltage of 3 kV, an aperture size of 30 µm, and a working distance (WD) of 1.4–3.2 mm. Samples were coated with a thin layer of gold/palladium (80/20 ratio), about 10 nm thick, to prevent charging due to the accumulation of static electric field when exposed to the electron beam. Furthermore, electron dispersive spectroscopy (EDS) was performed on the sample using the same instrument as the SEM. The setting parameters used for the SEM were an accelerating voltage of 20 kV, an aperture size of 60 µm, and a working distance (WD) of 5–9 mm.

### Reduction kinetics study

Thermogravimetry analysis under hydrogen gas (H_2_-TGA) was performed to investigate the reduction behaviour of the Sr_0.95_Ti_0.3_Fe_0.7−*x*_Cu_*x*_O_3−*γ*_ (*x* = 0–0.1) system. The samples were subjected to controlled heating at a heating rate of 5 °C min^−1^ in a 5% H_2_/Ar (95%) environment at 400, 600, and 800 °C. Each temperature step was maintained for a dwell time of 60 minutes, ensuring a comprehensive analysis. According to the literature, a regression fit of the weight loss data was performed using OriginPro Software to model the kinetic process.^32^

### Exsolved particle size analysis

ImageJ software was used for particle size analysis, with particle size distribution determined by Gaussian curve fitting in Origin Pro software. To determine the exsolved particle population density, particle size measurements were taken from three areas of each sample, all with the exact dimensions. The population density was then determined by dividing the number of particles by the area and averaging.

### Surface characterisation

The XPS analysis data were collected using the Kratos analytical system, which utilised a monochromatic Al Kα source. A filament current of 0.45 A and a filament bias of 1 V were used. Charge compensation was included in the data collection. Deconvolution of the core XPS spectra of the perovskite components was achieved using the KherveFitting software. All spectra deconvolution followed a specific spectra model in the KherveFitting Software library. A Voigt (area, *L*/*G*, *σ*) was used for the spectra of C 1s, O 1s, Ti 2p, and Sr 3d, while a mixed Gaussian–Lorentzian line shape (GL30) was used for Fe 2p. The GL30 function was adopted in this study because it is widely recommended for oxides in the literature.^47,48^ On the other hand, a mixed Gaussian–Lorentzian line shape (GL, as optimised during the fitting) was used for the spectra of CU 2p. Furthermore, for Cu 2p spectra, the work of Torress-Ochoa *et al.* guided most components and species identification according to BE.^49^

For the deconvolution of the high-resolution spectra (C 1s, O 1s, Ti 2p, Sr 3d, Fe 2p, and Cu 2p), background subtraction was performed using the Multi-region Smart algorithm implemented in the KherveFitting software. The Multi-region Smart combines Shirley and linear background models to account for both step-like changes in intensity near the photoemission edges and gradual linear drifts across wider energy windows, although the Tougaard-type backgrounds are often recommended in the literature for Fe and Cu core level spectra, as they can more accurately capture the extended inelastic loss features and multiplet-related structures that dominate these spectra.^50–52^ However, the additional parameterisation and assumptions regarding the energy-loss function introduced by Tougaard, particularly the background type, can complicate fitting when multiple overlapping species are present. Hence, the Multi-region Smart background was selected for a consistent and reproducible approach across all spectral regions, while effectively minimising residuals and avoiding over-parameterisation. This choice ensured uniform treatment of all core levels analysed, thereby facilitating direct comparison of relative peak areas between elements and oxidation states. Charge referencing or carbon correction was done for all the samples using adventitious C 1s (284.8 eV) as recommended in the literature.^48^

Surface elemental ratios were determined from high-resolution XPS by RSF-corrected quantification of Sr, Ti, Fe, Cu, and O. Carbonate contributions were excluded, oxygen was considered on a total-O basis (O-Lat + O-Vac + O-Surf), and shake-up satellites were omitted. The resulting atomic amounts were renormalised using a B-site normalisation scheme (Ti + Fe + Cu = 1) to yield elemental ratios relative to the transition-metal sublattice.

### X-ray absorption spectroscopy

The XAS analysis was performed at B18 beamline in Diamond Light Source. The spectra were obtained at the Cu and Fe K-edges, with a varying energy step from 0.25 to 0.35 eV using the average from 5 scans. Data analysis was carried out using the Athena software. XANES spectra were normalized by applying a linear fit to the pre-edge region and a second-order polynomial after the edge. The fractions of Cu species were determined by linear combination analysis −15 eV before and +30 eV after the edge using Cu foil, Cu_2_O, and CuO as reference samples for Cu (0), (i) and (ii), respectively, and the sample before exsolution. The EXAFS analysis consisted of *k*^2^-weighted Fourier transform of the data over the *k*-range from 1.9 to 11.5 Å^−1^. The samples consisted of quartz capillary tubes sealed by Kapton wool and filled with the Cu-doped (*x* = 0.1 mol of Cu) perovskite powder under a controlled atmosphere (4% H_2_/He) for exsolution at a ramp rate of 5 °C min^−1^. After exsolution, the spectra were collected under a pure He atmosphere at room temperature without any exposure to air.

### Electrochemical activity study

The electrochemical measurements were performed using a Metrohm Autolab PGSTAT302N electrochemical workstation in a three-electrode glass cell configuration. The working electrode (WE) was prepared by drop-casting a dispersion ink onto a 5 mm diameter glassy carbon (GC) electrode. The ink was prepared by dispersing 5 mg of perovskite oxide powder in a mixture of 250 µL isopropanol, 247.5 µL ultrapure water (Milli-Q, 18.2 MΩ), and 2.5 µL of a 5% Nafion solution. The solution was sonicated for 20 minutes to ensure homogeneity. A 20 µL aliquot of the ink was deposited on the GC electrode and dried in an oven at 60 °C for 15 minutes. Cyclic voltammetry experiments were conducted with a platinum mesh as the counter electrode and a reversible hydrogen electrode (RHE) as the reference electrode. The voltammograms were recorded at a sweep rate of 50 mV s^−1^ over a potential range from 1.4 V to −0.7 V *vs.* RHE. Blank profiles were recorded in 0.1 mol L^−1^ NaOH (Sigma-Aldrich®, 99.99%), NO_3_RR CVs were carried out in 0.1 mol L^−1^ NaOH with 1 mM NaNO_3_ (Labsynth®, 99.0%) as the nitrate source.

## Conclusion

This study systematically explored the impact of Cu doping on the structure, reduction kinetics, and exsolution behaviour of Sr_0.95_Ti_0.3_Fe_0.7−*x*_Cu_*x*_O_3−*γ*_ (*x*: 0–0.2) perovskites, aiming to optimise their properties for catalytic applications. Incorporating Cu significantly enhanced reduction kinetics, facilitating the controlled exsolution of Cu nanoparticles. XRD, Rietveld refinement, SEM, *in situ* XAS and XPS analyses confirmed the successful synthesis and exsolution process. SEM and particle size analysis revealed that reduction at 600 °C for 3 hours yielded the highest nanoparticle population density (∼650 particles per µm^2^), resulting in a higher density of active sites beneficial for catalytic performance. However, extended reduction times led to larger nanoparticles, presenting a trade-off between active site density and surface area availability. Furthermore, the *in situ* XAS, in conjunction with the XPS result, further confirmed that Cu is the primary exsolved species, showing the partial reduction of Cu^2+^ to Cu^0^ and the emergence of Cu–Cu coordination after exsolution, while the Fe environment remained largely unchanged. Overall, the combined electrochemical and XPS surface characterisation indicates that the exsolution temperatures significantly influenced the material's catalytic behaviour, with only samples treated at 400 °C and 600 °C showing NO_3_RR activity due to the presence of exsolved Cu nanoparticles. The 600 °C exsolved sample exhibited slightly lower activity than the 400 °C sample, which could be attributed to differences in particle size, morphology, population density, and composition. These results indicate a strong correlation between exsolution conditions and electrocatalytic activity, highlighting the trade-off between nanoparticle features and activity, where desirable performance requires balancing particle formation temperature with preserving a high surface area.

## Author contributions

Ubong A. Essien: investigation, data curation, formal analysis, simulation, visualization, funding acquisition, writing – review & editing, writing – original draft. Swathi P. Raju: investigation, formal analysis, writing – review & editing. Keyla T. Santos: investigation, formal analysis, writing – review & editing. Rafael A. Vicente: investigation, formal analysis, writing – review & editing. Chinyere Adaora Ekperechukwu: methodology. Francisco R. García-García: methodology, supervision, funding acquisition, writing – review & editing. Pablo S. Fernández: conceptualization, methodology, supervision, funding acquisition, writing – review & editing. Dragos Neagu: conceptualization, methodology, supervision, funding acquisition, writing – review & editing.

## Conflicts of interest

The authors declare that they have no known competing financial interests or personal relationships that could have appeared to influence the work reported in this paper.

## Supplementary Material

NA-008-D5NA00426H-s001

## Data Availability

The data supporting this article have been included as part of the supplementary information (SI). All crystal structures reported in this paper (CSD: 2519411–2519412, 2519413–2519414, 2519415–2519416, 2519417–2519418, 2519419, 2519420–2519421, 2519553, 2519554–2519555, 2519556–2519557, 2519558–2519559, and 2519560–2519561) are available on the CCDC database. [Clarification: Crystal structures whose CSD ID appears as a range indicate a perovskite structure and its corresponding secondary phase or exsolved phase (for reduced perovskite) structure]. Furthermore, the crystal information files (1525550, 1011148, and 4105681) used for Rietveld refinement in this study are available in the crystallographic open database at https://www.crystallography.net/cod/search.php. Supplementary information: additional Rietveld refinement data, SEM/SEM-EDS characterisation of nnaoparticle exsolution, detailed XPS analysis, particle size and population density measurements, and electrochemical reproducibility data supporting the main results. See DOI: https://doi.org/10.1039/d5na00426h.
