# Supplementary material for: Copper nanoparticle exsolution from Sr(Ti, Fe)O3 perovskites: material tuning and probing (electro)catalytic applicability
Source: Nanoscale Adv. 2025 Dec 10;8(6):1916–33. doi: 10.1039/d5na00426h (PMC12908656; doi:10.1039/d5na00426h)
Supplement: NA-008-D5NA00426H-s001 [file NA-008-D5NA00426H-s001.pdf]

Supplementary Information (SI) for Nanoscale Advances.
This journal is © The Royal Society of Chemistry 2025

Supplementary Information

Copper Nanoparticles Exsolution from Sr(Ti, Fe)O_3 Perovskites: Material Tuning and Probing (Electro)catalytic Applicability

Table of Contents

Note S1. Perovskite structural parameters and stoichiometry determined from the Rietveld refinement data.

Note S2. Exsolved nanoparticles population density and population density-error determination

Table S1. Summary of particle size analysis for the $Sr_{0.95}Ti_{0.3}Fe_{0.7}O_{3-\gamma}$ and $Sr_{0.95}Ti_{0.3}Fe_{0.6}Cu_{0.1}O_{3-\gamma}$

Note S3. Exsolution characteristics of the $Sr_{0.95}Ti_{0.3}Fe_{0.7}O_{3-\gamma}$ (undoped perovskite) revealed by SEM

Figure S1. SEM image showing limited exsolution in samples of $Sr_{0.95}Ti_{0.3}Fe_{0.7}O_{3-\gamma}$ reduced at different temperatures and no exsolution in the sintered (pristine) sample of $Sr_{0.95}Ti_{0.3}Fe_{0.6}Cu_{0.1}O_{3-\gamma}$ perovskite.

Note S4. Further information on the exsolution of $Sr_{0.95}Ti_{0.3}Fe_{0.6}Cu_{0.1}O_{3-\gamma}$ perovskite and the SEM-EDS result

Figure S2. Observed exsolution on $Sr_{0.95}Ti_{0.3}Fe_{0.6}Cu_{0.1}O_{3-\gamma}$ sample reduced at 600°C in 1 hour.

Table S2. The elemental composition of the $Sr_{0.95}Ti_{0.3}Fe_{0.6}Cu_{0.1}O_{3-\gamma}$ based on the SEM-EDS result

Table S3. Summary of the cell parameters from the Rietveld refinement of $Sr_{0.95}Ti_{0.3}Fe_{0.7-x}Cu_xO_{3-\gamma}$ (x: 0 – 0.2) reduced at 600°C and 1 hour

NOTE S5. XPS Results of the Perovskite Samples

Figure S3. Comparing high resolution core spectra of C1s, O1s, Ti2p, Fe2p and Sr3d for $Sr_{0.95}Ti_{0.3}Fe_{0.7}O_{3-\gamma}$ Sintered and Reduced (600 °C, 1hr) sample, after deconvolution.

Figure S4. Comparing high resolution core spectra of C1s, O1s, Ti2p, Sr3d, Fe2p and Cu2p, for $Sr_{0.95}Ti_{0.3}Fe_{0.7-x}Cu_xO_{3-\gamma}$ Reduced (400, 600, and 800 °C, 1hr) samples, after deconvolution.

Table S4. $Sr_{0.95}Ti_{0.3}Fe_{0.7}O_{3-\gamma}$ Sintered Components XPS analysis result summary

Table S5. $Sr_{0.95}Ti_{0.3}Fe_{0.7}O_{3-\gamma}$ Red-600 sample Components XPS analysis result summary

Table S6. $Sr_{0.95}Ti_{0.3}Fe_{0.6}Cu_{0.1}O_{3-\gamma}$ sintered components XPS analysis result summary

Table S7. $Sr_{0.95}Ti_{0.3}Fe_{0.6}Cu_{0.1}O_{3-\gamma}$ Red-400 components XPS analysis result summary

Table S8. $Sr_{0.95}Ti_{0.3}Fe_{0.6}Cu_{0.1}O_{3-\gamma}$ Red-600 components XPS analysis result summary

Table S9. $Sr_{0.95}Ti_{0.3}Fe_{0.6}Cu_{0.1}O_{3-\gamma}$ Red-800 components XPS analysis result summary

Table S10. Comparing the surface elemental ratio of the sintered perovskites from XPS to their nominal and calculated stoichiometric value determined from the Rietveld refinement of XRD data.
Figure S5. Comparing the surface elemental ratio (obtained from XPS) of the $Sr_{0.95}Ti_{0.3}Fe_{0.7-x}Cu_xO_{3-\gamma}$ perovskite system to the nominal (as-designed) and those obtained from XRD-Rietveld refinement data

Figure S6. Duplicate of NO_3RR measurements of 400°C and 600°C samples, certifying its reproductibility.

Note S1: Perovskite's structural parameters and stoichiometry determined from the Rietveld refinement data.

Table 1 of the main paper summarises the structural parameters for the $Sr_{0.95}Ti_{0.3}Fe_{0.6}Cu_{0.1}O_{3-\gamma}$ (x: 0 – 0.2) system, the actual Cu-doping amount determined according to Equation S3 (Equation 2 in the main paper), and the resulting perovskite stoichiometry.

The actual stoichiometry determination confirmed whether the synthesised perovskites possessed the intended A-site deficiency and B-site stoichiometry. The exact stoichiometry of the perovskites was determined considering the formation of a CuO secondary phase at the end of the solid-state reaction process. Hence, the sintered powder will be composed of 1 mol of $Sr_{0.95}Ti_{0.3}Fe_{0.7-(x-y)}Cu_{x-y}O_{3-\gamma}$ and y mol of CuO, as opposed to the designed perovskite stoichiometry, $Sr_{0.95}Ti_{0.3}Fe_{0.7-x}Cu_{x}O_{3-\gamma}$.

Given that m_A, m_B, and m_C are the respective masses of the designed perovskite, secondary phase and resulting perovskite phase after the synthesis process. Also, M_A, M_B, and M_C are the respective molar masses of the expected perovskite, secondary phase and resulting perovskite phase after the synthesis process. By letting K equal the weight fraction of the secondary phase after the synthesis process, an equation relating y to K can be derived as follows:

$$K = \frac{m_B}{m_B + m_C} = \frac{m_B}{m_A} \quad \text{(S1)}$$

Since $Amount = \frac{Mass}{Molar\ mass}$

Therefore,

$$m_B = yM_B \quad \text{(S2)}$$

Since 1 mol of the perovskite is formed, substituting **Equation S2** into **S1** and rearranging, we have that:

$$y = \frac{KM_A}{M_B} \quad \text{(S3)}$$

The amount of Cu metal, 'y', that goes into the formation of the CuO phase is determined from **Equation S3** using the value of K determined from Rietveld refinement of the respective perovskites powder XRD data.

The different values of y determined for all the perovskites in the $Sr_{0.95}Ti_{0.3}Fe_{0.6}Cu_{0.1}O_{3-\gamma}$ (x: 0.05 – 0.2) system was used to calculate the actual Cu-doping amount and stoichiometry of the final perovskites synthesised. The actual Cu-doping amount was obtained for each perovskite by subtracting y from x (**Equation S4**).

Hence,

$$Actual\ Cu\ doping\ amount = x - y \quad \text{(S4)}$$

Noting that for a given perovskite structure ABO_3, the B-site composition must sum up to 1, the optimised perovskite's stoichiometry was obtained by normalising the stoichiometry of each element in the perovskite by (1-y) according to **Equation S5**.

Hence,

$$Optimised\ Perovskite = Sr_{0.95/_{1-y}}\ Ti_{0.3/_{1-y}}\ Fe_{0.7-(x-y)/_{1-y}}\ Cu_{x-y/_{1-y}}\ O_{3-X/_{1-y}} \quad \text{(S5)}$$

Note S2: Exsolved nanoparticles population density and population density-error determination

To determine the exsolved particles' population density (PD), particle size measurements for each sample were taken from three different areas of the same dimensions. ImageJ software was used to analyze the particle size. The population density was then determined by dividing the number of particles by the area to have the number of particles/Area. The sample population density was then taken as an average of the three values obtained.

The error in the population density values was obtained as an absolute error. The errors were determined by taking the mean of the three population density values for each sample according to **Equation S6**, where N is the total number of population density values.

$$\bar{x} = \left(\frac{PD_1 + PD_2 + PD_3}{N}\right) \quad \text{(S6)}$$

The absolute error for each value was then taken according to **Equation S7.**

$$Ae = |PD - \bar{x}| \quad \text{(S7)}$$

The error in the population density was then taken as the individual errors according to **Equation S8.**

$$Error = \left(\frac{Ae_1 + Ae_2 + Ae_3}{N}\right) \quad \text{S8}$$

The results of the particle size analysis for the perovskite systems are presented in **Table S1**.

Table S1. Summary of particle size analysis for the $Sr_{0.95}Ti_{0.3}Fe_{0.7}O_{3-\gamma}$ and $Sr_{0.95}Ti_{0.3}Fe_{0.6}Cu_{0.1}O_{3-\gamma}$

Sample	Temperature (℃)	Time	Particle Size (nm)/ Shape	Density (Particle per µm²)	Comment
$Sr_{0.95}Ti_{0.3}Fe_{0.7}O_{3-\gamma}$	400	1 hour	No exsolution	-	No evidence of exsolution was observed.
	600		No exsolution	-	Exsolution is not evident by other methods, such as XRD and XPS.
	800		Difficult to imagine	-	Exsolution was evident but challenging to image.
$Sr_{0.95}Ti_{0.3}Fe_{0.6}Cu_{0.1}O_{3-\gamma}$	400		23 ± 4.7/ 12.7 ± 2.9 Spherical	118 ± 1	Two sets of particles were observed, one appearing to have grown at a different rate
	600		25.6 ± 4.3 Spherical	207 ± 2	Uniform sizes and evenly dispersed exsolved nanoparticles.
	800		37.6 ± 5.6 Cube-like and multifaceted	188 ± 4	Evenly dispersed multifaceted particles result from adjacent nanoparticles' intergrowth (joining).
	600	2 hours	26.1 ± 3.3 Spherical	520 ± 7	Dense and evenly dispersed spherical particles.
		3 hours	26.5 ± 5.2 Spherical	650 ± 7	Dense and evenly dispersed spherical particles
		4 hours	35.4 ± 5.7 Partly spherical	108 ± 3	Partly spherical particles with lots of offshoots due to the joining of adjacent nanoparticles during particle growth.

Note S3: Exsolution characteristics of the $Sr_{0.95}Ti_{0.3}Fe_{0.7}O_{3-\gamma}$ (undoped perovskite) revealed by SEM

The undoped perovskite, $Sr_{0.95}Ti_{0.3}Fe_{0.7}O_{3-\gamma}$, exhibited limited exsolution capability. The sample reduced at 400 ℃ in 1 hour (**Figure S1a**) showed no exsolution. For the samples reduced at 600 ℃ in 1 hour (**Figure S1b**), the presence of structures resembling exsolved nanoparticles, which appeared not to be uniformly dispersed throughout the sample, was observed. However, based on the XRD, Rietveld refinement, and XPS results, we argue that the observed particles at 600 ℃ could be a possible segregation instead of exsolved Fe nanoparticles. On the other hand, the sample reduced at 800℃ and 1 hour (**Figure S1c**) was extremely difficult to image, signifying that the exsolved particle sizes could have been well below the SEM detection limit. See **Figure 3** of the main article for the SEM of the unreduced sample of $Sr_{0.95}Ti_{0.3}Fe_{0.7}O_{3-\gamma}$ and $Sr_{0.95}Ti_{0.3}Fe_{0.6}Cu_{0.1}O_{3-\gamma}$ perovskite at µm scale. **Figure S1d** shows the pristine sample of $Sr_{0.95}Ti_{0.3}Fe_{0.6}Cu_{0.1}O_{3-\gamma}$ perovskite before reduction, highlighting its similarity with the undoped perovskite reduced at 400 ℃.

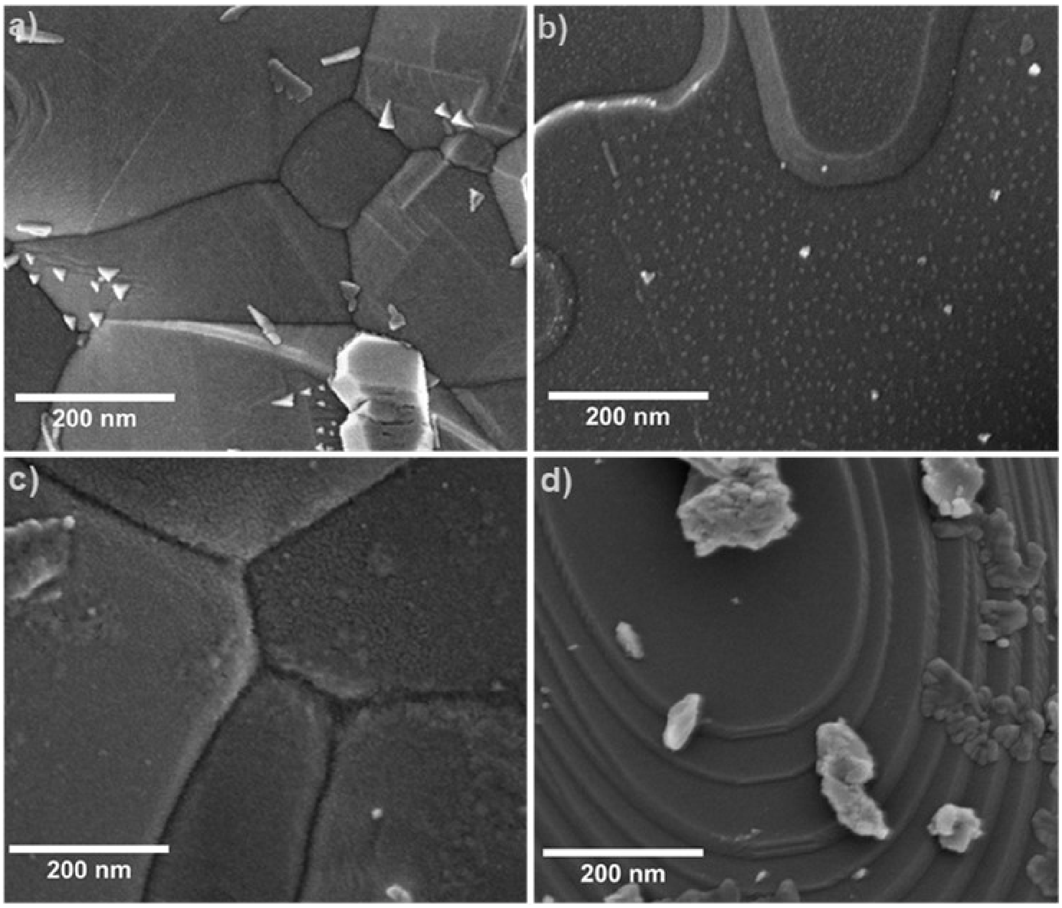

Figure S1. SEM image showing limited exsolution in samples of $Sr_{0.95}Ti_{0.3}Fe_{0.7}O_{3-\gamma}$ reduced at different temperatures and no exsolution in the sintered (pristine) sample of $Sr_{0.95}Ti_{0.3}Fe_{0.6}Cu_{0.1}O_{3-\gamma}$ perovskite. a) no observed exsolution in $Sr_{0.95}Ti_{0.3}Fe_{0.7}O_{3-\gamma}$ reduced at 400 °C in 1 hour; b) possible exsolution in $Sr_{0.95}Ti_{0.3}Fe_{0.7}O_{3-\gamma}$ sample reduced at 600°C in 1 hour; c) difficult to image exsolution in $Sr_{0.95}Ti_{0.3}Fe_{0.7}O_{3-\gamma}$ sample reduced 800 °C in 1 hour; and d) pristine sample of $Sr_{0.95}Ti_{0.3}Fe_{0.6}Cu_{0.1}O_{3-\gamma}$ perovskite before reduction.

Note S4: Further information on the exsolution of $Sr_{0.95}Ti_{0.3}Fe_{0.6}Cu_{0.1}O_{3-\gamma}$ perovskite and the SEM-EDS result.

The exsolution capability of the $Sr_{0.95}Ti_{0.3}Fe_{0.6}Cu_{0.1}O_{3-\gamma}$ perovskite system was further studied by SEM-EDS, revealing the even dispersion of the exsolved nanoparticles and the closeness of the perovskite's surface composition to the designed stoichiometry. The image of the perovskite taken during SEM-EDS and a comparison of the XRD pattern of the before and after exsolution is presented in **Figure S2. Table S2** presents the compositional information obtained from the SEM-EDS. Finally, **Table S3** presents the cell parameters and the change in cell parameters of the perovskite systems before and after exsolution for all the Cu-doping variations.

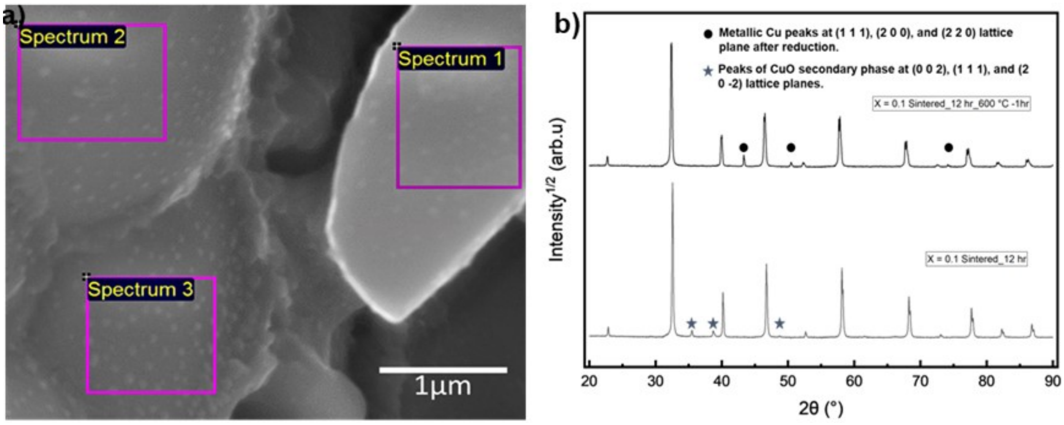

Figure S2. Observed exsolution on $Sr_{0.95}Ti_{0.3}Fe_{0.6}Cu_{0.1}O_{3-\gamma}$ sample reduced at 600°C in 1 hour. a) SEM-EDS image highlighting the surface composition, even dispersion of exsolved Cu nanoparticles, and b) XRD pattern comparing the peaks of the sintered sample to those of the reduced sample.

Table S2. The elemental composition of the $Sr_{0.95}Ti_{0.3}Fe_{0.6}Cu_{0.1}O_{3-\gamma}$ based on SEM-EDS result

Spectrum	In stats.	O	Ti	Fe	Cu	Sr	Total
Spectrum 1	Yes	27.92	8.25	18.92	1.6	43.31	100
Spectrum 2	Yes	17.22	10.55	26.41	1.42	44.41	100
Spectrum 3	Yes	18.82	10.61	27.44	1.86	41.28	100
Mean		21.32	9.81	24.26	1.62	43	100
Std. deviation		5.77	1.35	4.65	0.22	1.59	
Max.		27.92	10.61	27.44	1.86	44.41	
Min.		17.22	8.25	18.92	1.42	41.28	
Amount (mol)		2.47	0.38	0.81	0.05	0.91	

Table S3. Summary of the cell parameters from the Rietveld refinement of $Sr_{0.95}Ti_{0.3}Fe_{0.7-x}Cu_xO_{3-\gamma}$ (x: 0 – 0.2) reduced at 600°C and 1 hour

Perovskite/ Processing	Cell parameter (Å)		Change in Cell Parameter after reduction (Å)	Percentage Change in Cell Parameter (%)
	Sintered	Reduced		
x = 0	3.887	3.906	0.019	0.5
x = 0.05	3.885	3.897	0.012	0.3
x = 0.1	3.882	3.901	0.019	0.5
x = 0.15	3.881	3.908	0.027	0.7
x = 0.2	3.878	3.907	0.029	0.7

Note S5: XPS Results of the Perovskite Samples

Figure S3 and **S4** presents the high-resolution core-level XPS spectra deconvolution result for the perovskite samples. On the other hand, **Table S4 – S9** presents the summary of the deconvoluted high-resolution XPS core-level spectras (C1s, O1s, Ti2p, Sr3d, Fe2p and Cu2p) for the different perovskite samples. For all the perovskite samples, the **cross-elemental surface atomic percentage (CESAP)** were determined on the following basis: Total-O (considering the contributions of all O1s species), $SrCO_3$ excluded (all contributions of C1s and adsorbed carbonates excluded), all satellite and shake-up contributions (Fe, Cu, and Ti species satellites) excluded, and B-site normalisation.

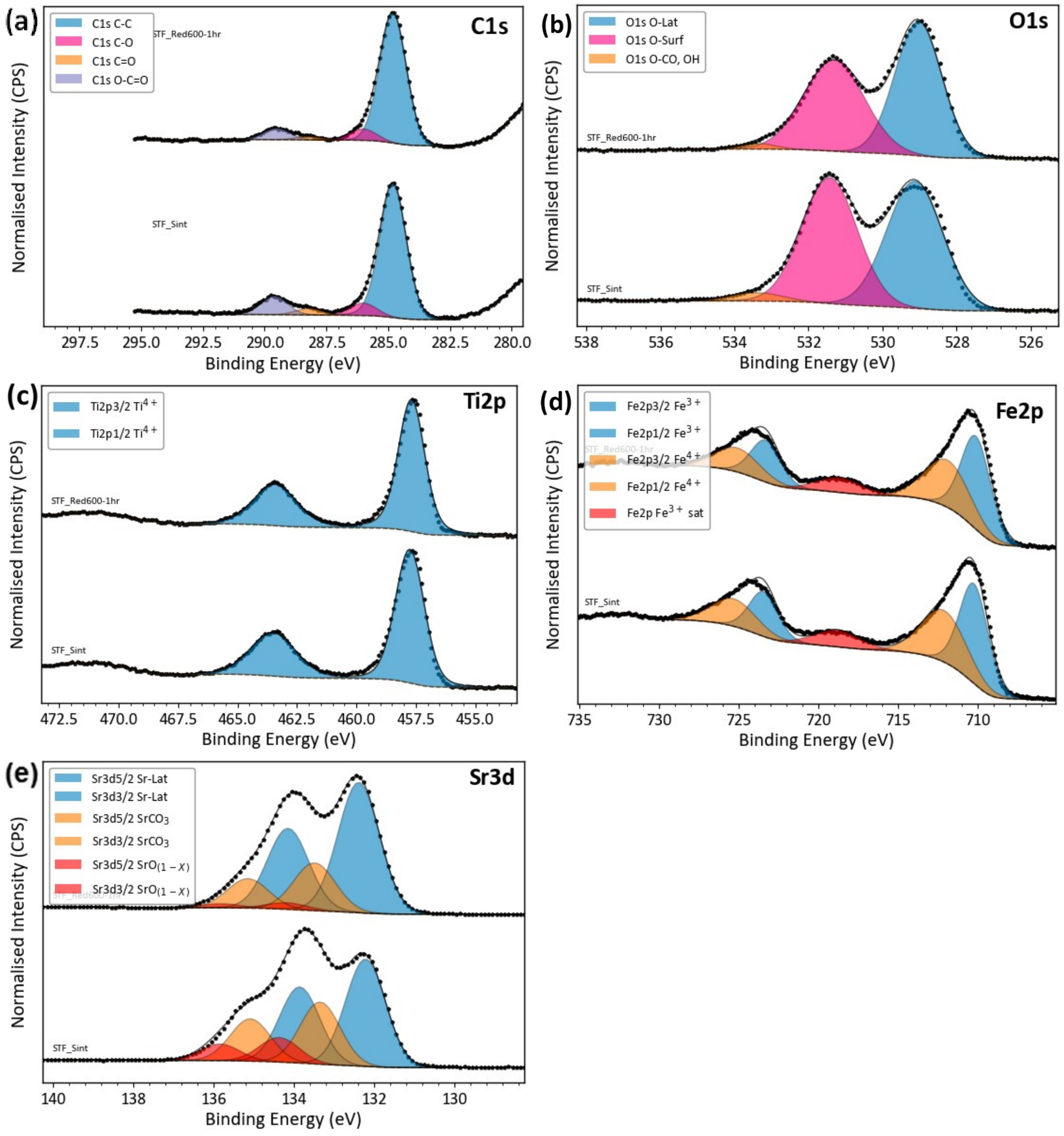

Figure S3. Comparing high resolution core spectra of C1s, O1s, Ti2p, Fe2p and Sr3d for $Sr_{0.95}Ti_{0.3}Fe_{0.7}O_{3-\gamma}$ Sintered and Reduced (600 ℃, 1hr) sample, after deconvolution. (a) C1s spectra deconvoluted into 4 components (C-C, C-O, C=C; and O-C=O); (b) O1s spectra deconvoluted into 3 components (O-Lat, O-Surface, and O-CO/O-OH); (c) Ti2p deconvoluted into 1 component (Ti^{4+} and Ti^{4+} doublet); (d) Fe2p spectra deconvoluted into 2 components and satellite peak (Fe^{3+}/Fe^{3+} doublet, Fe^{4+}/Fe^{4+} doublet, and a Fe^{3+} satalite peak]; and (e) Sr3d spectra deconvoluted into 3 components (Sr-Lat/Sr-Lat doublet, $SrCO_3$/$SrCO_3$ doublet, and $SrO_{(1-X)}$/ $SrO_{(1-X)}$ doublet).

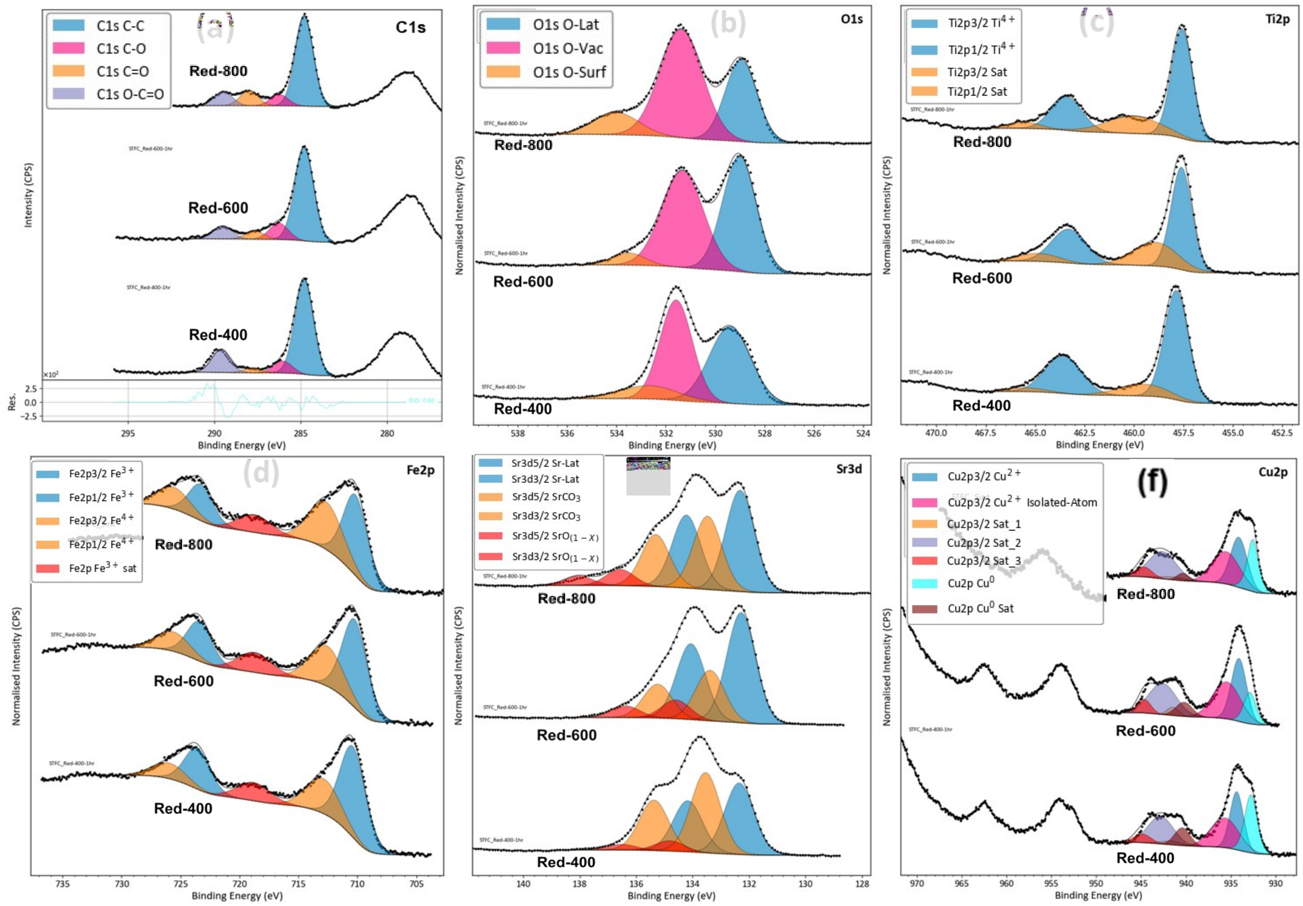

Figure S4. Comparing high resolution core spectra of C1s, O1s, Ti2p, Sr3d, Fe2p and Cu2p, for $Sr_{0.95}Ti_{0.3}Fe_{0.7-x}Cu_xO_{3-γ}$ Reduced (400, 600, and 800 ℃, 1hr) samples, after deconvolution. (a) C1s spectra deconvoluted into four components (C-C, C-O, C=C; and O-C=O) (b) O1s spectra deconvoluted into 3 components (O-Lat, O-Surface, and O-CO/O-OH); (c) Ti2p deconvoluted into 1 component (Ti^{4+}/Ti^{4+} doublet) and two additional satellite peaks; (d) Fe2p spectra deconvoluted into 2 components and 1 satellite peak (Fe^{3+}/Fe^{3+} doublet, Fe^{4+}/Fe^{4+} doublet, and Fe^{3+} satalite peak]; (e) Sr3d spectra deconvoluted into 3 components (Sr-Lat/Sr-Lat doublet, $SrCO_3$/$SrCO_3$ doublet, and $SrO_{(1-X)}$/ $SrO_{(1-X)}$ doublet); and (f) Cu2p, deconvoluted into 3 components and 4 satellite peaks (Cu^{2+}, Cu^{2+} Isolated atom, Cu^0, Satellite peak 1, 2, and 3, and Cu^0 satellite peak).

Table S4. $Sr_{0.95}Ti_{0.3}Fe_{0.7}O_{3-γ}$ Sintered Components XPS analysis result summary

Spectra	Species	Position	FWHM	Area	RSF	Atomic %	CESAP %
C 1s	C-C	284.80	1.26	170.61	0.28	77.79	
	C-O	286.00	1.26	15.91	0.28	7.25	
	C=O	288.20	1.26	8.68	0.28	3.96	
	O-C=O	289.60	1.26	24.13	0.28	11.00	
O 1s	O-Lat	529.13	1.79	112.41	0.78	49.12	58.03
	O- Surf	531.43	1.76	109.65	0.78	47.91	
	O-CO, OH	533.40	1.63	6.81	0.78	2.97	
Ti 2p	$2p_{3/2}$ Ti^{4+}	457.75	1.44	19.32	1.34	48.92	9.24
	$2p_{1/2}$ Ti^{4+}	463.50	2.27	21.32	0.66	51.08	
Sr 3d	$3d_{5/2}$ Sr-Lat	132.21	1.17	30.38	1.09	27.37	24.72
	$3d_{3/2}$ Sr-Lat	133.85	1.18	31.45	0.75	28.38	
	$3d_{5/2}$ $SrCO_3$	133.34	1.18	17.7	1.09	15.94	
	$3d_{3/2}$ $SrCO_3$	135.08	1.18	17.5	0.75	15.79	
	$3d_{5/2}$ $SrO_{(1-x)}$	134.36	1.18	7	1.09	6.31	
	$3d_{3/2}$ $SrO_{(1-x)}$	135.84	1.18	6.88	0.75	6.21	
Fe 2p	Fe^{3+} $2p_{3/2}$	710.22	2.22	13.71	1.95	26.83	8.01
	Fe^{3+} $2p_{1/2}$	723.42	2.22	14.24	1.01	23.61	
	Fe^{4+} $2p_{3/2}$	712.11	3.50	11.2	1.95	21.92	
	Fe^{4+} $2p_{1/2}$	725.31	3.50	13.76	1.01	22.82	
	Fe^{3+} sat	718.80	3.50	2.6	2.96	4.83	

Table S5. $Sr_{0.95}Ti_{0.3}Fe_{0.7}O_{3-\gamma}$ Red-600 sample Components XPS analysis result summary

Spectra	Species	Position	FWHM	Area	RSF	Atomic %	CESAP %
C 1s	C-C	284.80	1.27	152.24	0.28	82.92	
	C-O	286.00	1.26	13.35	0.28	7.27	
	C=O	288.20	1.26	4.23	0.28	2.30	
	O-C=O	289.52	1.26	13.8	0.28	7.51	
O 1s	O-Lat	529.03	1.50	109.46	0.78	52.54	56.19
	O- Surf	531.31	1.93	95.7	0.78	45.93	
	O-CO, OH	533.33	1.53	3.19	0.78	1.53	
Ti 2p	$2p_{3/2}$ Ti^{4+}	457.68	1.31	20.04	1.34	48.86	10.21
	$2p_{1/2}$ Ti^{4+}	463.42	2.15	22.16	0.66	51.14	
Sr 3d	$3d_{5/2}$ Sr-Lat	132.38	1.21	40.94	1.09	36.98	29.45
	$3d_{3/2}$ Sr-Lat	134.14	1.21	37.07	0.75	33.55	
	$3d_{5/2}$ $SrCO_3$	133.48	1.21	15.06	1.09	13.60	
	$3d_{3/2}$ $SrCO_3$	135.14	1.21	13.5	0.75	12.22	
	$3d_{5/2}$ $SrO_{(1-x)}$	134.17	1.21	2.12	1.09	1.92	
	$3d_{3/2}$ $SrO_{(1-x)}$	135.81	1.21	1.9	0.75	1.72	
Fe 2p	Fe^{3+} $2p_{3/2}$	710.09	2.28	12.8	1.95	27.74	7.67
	Fe^{3+} $2p_{1/2}$	723.29	2.28	13.29	1.01	24.41	
	Fe^{4+} $2p_{3/2}$	711.88	3.50	9.97	1.95	21.61	
	Fe^{4+} $2p_{1/2}$	725.08	3.50	11.76	1.01	21.61	
	Fe^{3+} sat	718.67	3.50	2.25	2.96	4.63	

Table S6. $Sr_{0.95}Ti_{0.3}Fe_{0.6}Cu_{0.1}O_{3-\gamma}$ sintered components XPS analysis result summary

Spectra	Species	Position	FWHM	Area	RSF	Atomic %	CESAP %
C 1s	C-C	284.80	1.28	539.27	0.28	75.91	
	C-O	286.00	1.28	61.97	0.28	8.72	
	C=O	288.17	1.28	21.63	0.28	3.04	
	O-C=O	289.60	1.28	87.58	0.28	12.33	
O 1s	O-Lat	529.07	1.86	368.96	0.78	43.71	61.76
	O-Surf	531.46	1.81	451.69	0.78	53.52	
	O-CO, OH	533.50	1.47	23.35	0.78	2.77	
Ti 2p	$2p_{3/2}$ Ti^{4+}	457.73	1.53	58.76	1.34	47.88	8.03
	$2p_{1/2}$ Ti^{4+}	463.46	2.44	63.95	0.66	52.12	
Sr 3d	$3d_{5/2}$ Sr-Lat	132.10	1.12	101.81	1.09	23.68	21.12
	$3d_{3/2}$ Sr-Lat	134.94	1.11	102.84	0.75	23.92	
	$3d_{5/2}$ $SrCO_3$	133.20	1.11	63.56	1.09	14.78	
	$3d_{3/2}$ $SrCO_3$	134.92	1.11	64.59	0.75	15.02	
	$3d_{5/2}$ $SrO_{(1-x)}$	133.67	1.07	47.79	1.09	11.12	
	$3d_{3/2}$ $SrO_{(1-x)}$	135.57	1.07	49.35	0.75	11.48	
Fe 2p	Fe^{3+} $2p_{3/2}$	710.18	2.35	61.77	1.95	29.17	8.48
	Fe^{3+} $2p_{1/2}$	723.38	2.35	54.35	1.01	25.67	
	Fe^{4+} $2p_{3/2}$	712.30	3.50	42.61	1.95	20.12	
	Fe^{4+} $2p_{1/2}$	725.48	3.50	41.62	1.01	19.65	
	Fe^{3+} sat	718.75	3.50	11.41	2.96	5.39	
Cu 2p	Cu^{2+} $2p_{3/2}$	933.78	2.28	18.03	3.51	30.56	0.61
	Cu^{2+} $2p_{3/2}$ Iso-atom	935.10	3.70	19.22	3.51	32.57	
	Cu $2p_{3/2}$ Sat1	942.14	1.78	1.77	3.51	2.99	
	Cu $2p_{3/2}$ Sat2	942.28	3.70	16.45	3.51	27.87	
	Cu $2p_{3/2}$ Sat3	944.33	1.58	3.54	3.51	6.00	

Table S7. $Sr_{0.95}Ti_{0.3}Fe_{0.6}Cu_{0.1}O_{3-\gamma}$ 400 °C reduced sample components' XPS analysis result summary

Spectra	Species	Position	FWHM	Area	RSF	Atomic %	CESAP %
C 1s	C-C	284.80	1.27	369.72	0.28	70.43	
	C-O	286.05	1.26	47.07	0.28	8.96	
	C=O	287.78	1.26	17.95	0.28	3.42	
	O-C=O	289.60	1.26	90.22	0.28	17.19	
O 1s	O-Lat	529.40	1.99	354.52	0.78	44.25	60.26
	O-Surf	531.58	1.54	359.76	0.78	44.91	
	O-CO, OH	532.66	2.79	86.8	0.78	10.84	
Ti 2p	$2p_{3/2}$ Ti^{4+}	457.86	1.44	50.4	1.34	41.70	6.96
	$2p_{1/2}$ Ti^{4+}	463.59	2.18	53.48	0.66	44.25	
	$2p_{3/2}$ Sat	459.52	3.02	11.42	1.34	9.45	

	$2p_{1/2}$ Sat	465.41	2.20	5.56	0.66	4.61	
Sr 3d	$3d_{5/2}$ Sr-Lat	132.35	1.23	84.18	1.09	22.46	24.67
	$3d_{3/2}$ Sr-Lat	134.17	1.22	87.58	0.75	23.36	
	$3d_{5/2}$ $SrCO_3$	133.54	1.22	94.4	1.09	25.18	
	$3d_{3/2}$ $SrCO_3$	135.37	1.23	86.41	0.75	23.05	
	$3d_{5/2}$ $SrO_{(1-x)}$	134.81	1.22	12.28	1.09	3.28	
	$3d_{3/2}$ $SrO_{(1-x)}$	136.51	1.22	9.99	0.75	2.66	
Fe 2p	Fe^{3+} $2p_{3/2}$	710.38	2.68	61.09	1.95	33.34	7.46
	Fe^{3+} $2p_{1/2}$	723.58	2.68	53.75	1.01	29.34	
	Fe^{4+} $2p_{3/2}$	712.86	3.50	30.76	1.95	16.79	
	Fe^{4+} $2p_{1/2}$	725.95	3.50	27.07	1.01	14.78	
	Fe^{3+} sat	718.99	3.50	10.53	2.96	5.75	
Cu 2p	Cu^{2+} $2p_{3/2}$	934.32	1.78	15.77	3.51	24.11	0.66
	Cu^{2+} $2p_{3/2}$ Iso-atom	935.64	3.70	16.83	3.51	25.72	
	Cu $2p_{3/2}$ Sat1	941.68	2.73	1.54	3.51	2.36	
	Cu $2p_{3/2}$ Sat2	942.82	3.38	14.4	3.51	22.01	
	Cu $2p_{3/2}$ Sat3	944.87	2.30	3.1	3.51	4.74	
	Cu^0 $2p_{3/2}$	932.74	1.72	10	5.32	15.28	
	Cu^0 $2p_{3/2}$ Sat	940.39	2.08	3.78	5.32	5.78	

Table S8. $Sr_{0.95}Ti_{0.3}Fe_{0.6}Cu_{0.1}O_{3-\gamma}$ 600 °C reduced sample components' XPS analysis result summary

Spectra	Species	Position	FWHM	Area	RSF	Atomic %	CESAP %
C 1s	C-C	284.80	1.28	377.07	0.28	71.05	
	C-O	286.27	1.28	66.82	0.28	12.59	
	C=O	287.67	1.28	33.77	0.28	6.36	
	O-C=O	289.51	1.28	53.07	0.28	10.00	
O 1s	O-Lat	529.02	1.61	359.88	0.78	46.70	58.47
	O- Surf	531.32	1.99	373.83	0.78	48.51	
	O-CO, OH	533.51	1.54	36.96	0.78	4.80	
Ti 2p	$2p_{3/2}$ Ti^{4+}	457.60	1.25	47.04	1.34	34.18	6.80
	$2p_{1/2}$ Ti^{4+}	463.31	2.06	52.63	0.66	38.25	
	$2p_{3/2}$ Sat	458.92	2.66	23.38	1.34	16.99	
	$2p_{1/2}$ Sat	464.82	2.42	14.56	0.66	10.58	
Sr 3d	$3d_{5/2}$ Sr-Lat	132.28	1.21	123.29	1.09	30.91	26.52
	$3d_{3/2}$ Sr-Lat	134.06	1.21	123.53	0.75	30.97	
	$3d_{5/2}$ $SrCO_3$	133.38	1.21	56.59	1.09	14.19	
	$3d_{3/2}$ $SrCO_3$	135.24	1.21	55.14	0.75	13.83	
	$3d_{5/2}$ $SrO_{(1-x)}$	134.59	1.21	21.15	1.09	5.30	
	$3d_{3/2}$ $SrO_{(1-x)}$	136.38	1.21	19.11	0.75	4.79	
Fe 2p	Fe^{3+} $2p_{3/2}$	710.22	2.58	63.09	1.95	30.41	7.44
	Fe^{3+} $2p_{1/2}$	723.40	2.58	55.51	1.01	26.76	
	Fe^{4+} $2p_{3/2}$	712.59	3.50	39.1	1.95	18.85	
	Fe^{4+} $2p_{1/2}$	725.61	3.50	37.04	1.01	17.85	
	Fe^{3+} sat	718.70	3.50	12.73	2.96	6.13	
Cu 2p	Cu^{2+} $2p_{3/2}$	934.47	2.05	20.59	3.51	22.13	0.78

	Cu^{2+} $2p_{3/2}$ Iso-atom	935.79	3.7	21.97	3.51	23.61	
	Cu $2p_{3/2}$ Sat1	941.83	3.29	2.01	3.51	2.16	
	Cu $2p_{3/2}$ Sat2	943.05	3.0	18.8	3.51	20.20	
	Cu $2p_{3/2}$ Sat3	945.02	2.4	4.05	3.51	4.35	
	Cu^0 $2p_{3/2}$	933.28	2.16	5.66	5.32	17.67	
	Cu^0 $2p_{3/2}$ Sat	940.74	2.48	3.37	5.32	9.88	

Table S9. $Sr_{0.95}Ti_{0.3}Fe_{0.6}Cu_{0.1}O_{3-\gamma}$ 800 °C reduced sample components' XPS analysis result summary

Spectra	Species	Position	FWHM	Area	RSF	Atomic %	CESAP %
C 1s	C-C	284.80	1.23	365.06	0.28	68.85	
	C-O	286.25	1.23	45.65	0.28	8.83	
	C=O	287.99	1.23	59.59	0.28	11.52	
	O-C=O	289.48	1.23	55.84	0.28	10.80	
O 1s	O-Lat	528.95	1.53	231.52	0.78	31.65	60.11
	O- Surf	531.38	2.05	411.3	0.78	56.22	
	O-CO, OH	534.01	2.14	88.76	0.78	12.13	
Ti 2p	$2p_{3/2}$ Ti^{4+}	457.58	1.27	37.49	1.34	39.68	5.13
	$2p_{1/2}$ Ti^{4+}	463.41	1.86	34.39	0.66	36.42	
	$2p_{3/2}$ Sat	460.03	3.21	15.9	1.34	16.83	
	$2p_{1/2}$ Sat	465.55	1.85	6.68	0.66	7.07	
Sr 3d	$3d_{5/2}$ Sr-Lat	132.32	1.20	102.33	1.09	26.10	28.35
	$3d_{3/2}$ Sr-Lat	134.22	1.19	107.68	0.75	27.46	
	$3d_{5/2}$ $SrCO_3$	133.47	1.19	74.44	1.09	18.98	
	$3d_{3/2}$ $SrCO_3$	135.31	1.19	76.88	0.75	19.61	
	$3d_{5/2}$ $SrO_{(1-x)}$	136.57	1.19	16.78	1.09	4.28	
	$3d_{3/2}$ $SrO_{(1-x)}$	138.03	1.19	14	0.75	3.57	
Fe 2p	Fe^{3+} $2p_{3/2}$	710.15	2.55	34.94	1.95	27.64	5.65
	Fe^{3+} $2p_{1/2}$	723.28	2.55	30.74	1.01	24.32	
	Fe^{4+} $2p_{3/2}$	712.64	3.50	28.31	1.95	22.39	
	Fe^{4+} $2p_{1/2}$	725.59	3.50	25.53	1.01	20.20	
	Fe^{3+} sat	718.74	3.50	6.89	2.96	5.45	
Cu 2p	Cu^{2+} $2p_{3/2}$	934.17	2.19	17.5	3.51	25.65	0.76
	Cu^{2+} $2p_{3/2}$ Iso-atom	935.59	3.7	18.67	3.51	27.36	
	Cu $2p_{3/2}$ Sat1	941.62	3.7	1.71	3.51	2.51	
	Cu $2p_{3/2}$ Sat2	942.77	3.7	15.98	3.51	23.42	
	Cu $2p_{3/2}$ Sat3	944.82	2.46	3.44	3.51	5.05	
	Cu^0 $2p_{3/2}$	932.57	1.52	8.05	5.32	13.46	
	Cu^0 $2p_{3/2}$ Sat	940.41	1.62	1.26	5.32	2.56	

Table S10. Comparing the surface elemental ratio of the sintered perovskites from XPS to their nominal and calculated stoichiometric value determined from the Rietveld refinement of XRD data.

Perovskite	Processing Condition		Elemental Ratio			
		**Sr**	**TI**	**Fe**	**O**	
$Sr_{0.95}Ti_{0.3}Fe_{0.7}O_{3-\gamma}$	**Nominal**	0.95	0.3	0.7	(3-γ)	
	**Calculated**	0.95	0.3	0.7	(3-γ)	
	**Sintered**	1.43	0.54	0.46	3.36	
	**Red-600**	1.45	0.57	0.43	3.14	
$Sr_{0.95}Ti_{0.3}Fe_{0.6}Cu_{0.1}O_{3-\gamma}$		**Sr**	**TI**	**Fe**	**Cu**	**O**
	**Nominal**	0.95	0.3	0.60	0.1	(3-γ)
	**Calculated**	1.04	0.33	0.65	0.02	3-γ
	**Sintered**	1.23	0.47	0.5	0.04	3.61
	**Red-400**	1.63	0.46	0.5	0.04	4.00
	**Red-600**	1.76	0.45	0.49	0.05	3.89
	**Red-800**	2.46	0.37	0.49	0.07	5.21

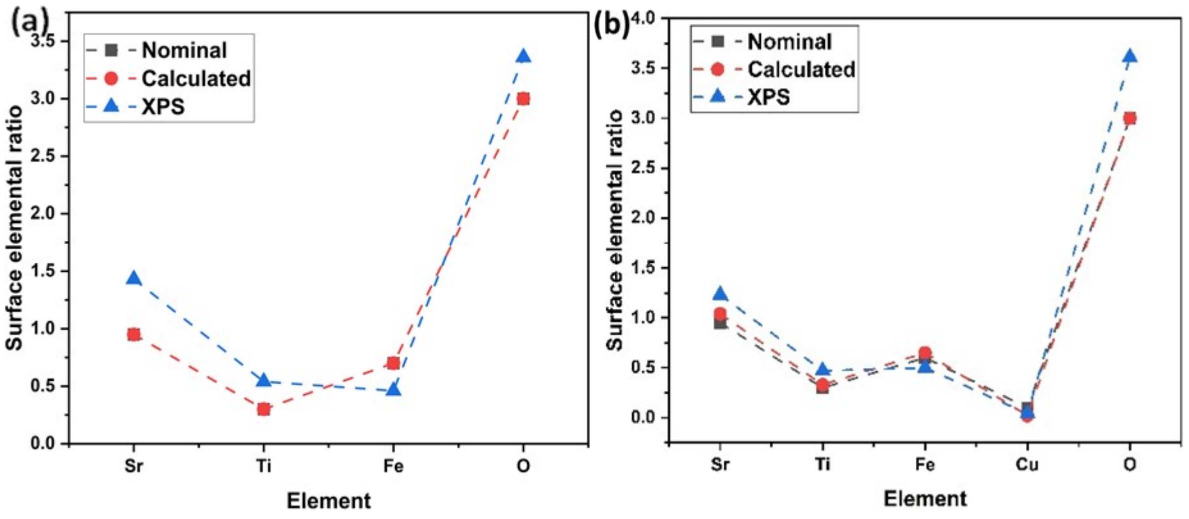

Figure S5. Comparing the surface elemental ratio (obtained from XPS) of the $Sr_{0.95}Ti_{0.3}Fe_{0.7-x}Cu_xO_{3-\gamma}$ perovskite system to the nominal (as-designed) and those obtained from XRD-Rietveld refinement data: (a) comparison for the x = 0 (undoped) sample; (b) comparison for the x = 0.1 sintered sample. Result shows the deviation of samples from the nominal stoichiometry and the ability of the XPS approach to determine possible surface reconstruction.

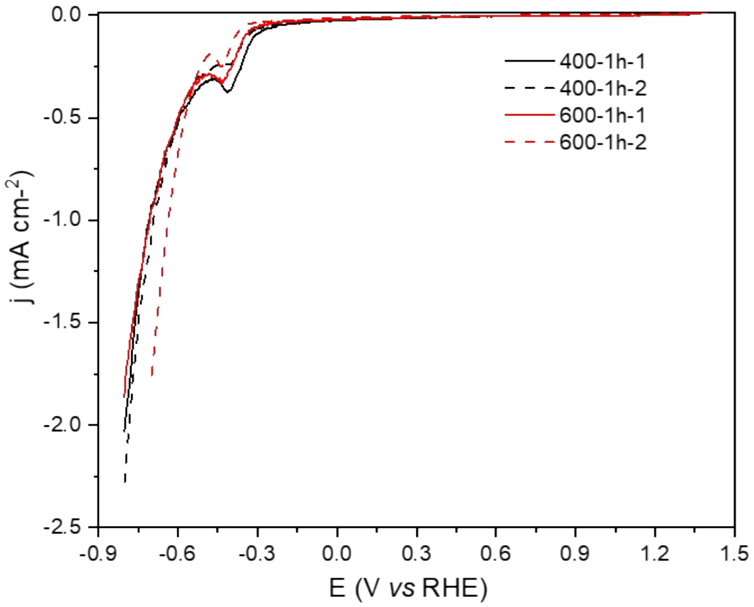

Figure S6. Duplicate of cathodic sweep of CVs for $Sr_{0.95}Ti_{0.3}Fe_{0.6}Cu_{0.1}O_{3-\gamma}$, after exsolution at 400°C, and 600 °C for 1h in 0.1 M NaOH and 1 mM $NaNO_3$ at 50 mVs^{-1}. Each measurement was carried independently.
